# Radiomics and radiogenomics: extracting more information from medical images for the diagnosis and prognostic prediction of ovarian cancer

**DOI:** 10.1186/s40779-024-00580-1

**Published:** 2024-12-14

**Authors:** Song Zeng, Xin-Lu Wang, Hua Yang

**Affiliations:** Department of Ultrasound, Shengjing Hospital of China Medical University, Shenyang, 110004 China

**Keywords:** Radiomics, Radiogenomics, Machine learning, Deep learning, Ovarian cancer

## Abstract

Ovarian cancer (OC) remains one of the most lethal gynecological malignancies globally. Despite the implementation of various medical imaging approaches for OC screening, achieving accurate differential diagnosis of ovarian tumors continues to pose significant challenges due to variability in image performance, resulting in a lack of objectivity that relies heavily on the expertise of medical professionals. This challenge can be addressed through the emergence and advancement of radiomics, which enables high-throughput extraction of valuable information from conventional medical images. Furthermore, radiomics can integrate with genomics, a novel approach termed radiogenomics, which allows for a more comprehensive, precise, and personalized assessment of tumor biological features. In this review, we present an extensive overview of the application of radiomics and radiogenomics in diagnosing and predicting ovarian tumors. The findings indicate that artificial intelligence methods based on imaging can accurately differentiate between benign and malignant ovarian tumors, as well as classify their subtypes. Moreover, these methods are effective in forecasting survival rates, treatment outcomes, metastasis risk, and recurrence for patients with OC. It is anticipated that these advancements will function as decision-support tools for managing OC while contributing to the advancement of precision medicine.

## Background

Ovarian cancer (OC) ranks as the eighth most prevalent and fifth most lethal malignancy among women worldwide. Over 300,000 women are diagnosed with OC annually, resulting in approximately 152,000 fatalities OC each year, highlighting the significant threat this illness poses to the health and lives of women [1]. OC is not a single disease; it can be categorized into at least 6 distinct histological subtypes according to the World Health Organization (WHO) 2020 classification of female reproductive organ malignancies. The majority of these fall under epithelial-mesenchymal tumors, including serous tumors, mucinous tumors, endometrioid tumors, clear cell tumors, seromucinous tumors, and Brenner tumors [2]. Among these types, high-grade serous ovarian carcinoma (HGSOC) is the most common histological subtype, accounting for approximately 90% of OC, and has the poorest prognosis compared to its counterpart low-grade serous ovarian carcinoma (LGSOC) [3]. Standard treatments for newly diagnosed OC typically involve cytoreductive surgery followed by platinum-based chemotherapy; however, prognostic outcomes remain unsatisfactory [4]. This lack of improvement is attributed to intertumoral and intratumoral heterogeneities within OC that hinder comprehensive research on tumor behavior and result in imprecise diagnosis and inadequate predictions regarding disease progression [5, 6]. Furthermore, a study has revealed significant disparities in early versus late survival rates for OC [7], underscoring the critical importance of developing effective early diagnosis and prognosis prediction methods.

Imaging plays a pivotal role in the assessment and management of OC [8]. Traditional imaging methods, such as ultrasound, computed tomography (CT), positron emission tomography/CT (PET/CT), and magnetic resonance imaging (MRI), have been extensively employed for the diagnosis and evaluation of ovarian tumors due to their convenience and non-invasive nature [8–11]. Furthermore, serum biomarkers offer a cost-effective approach to predicting OC. Currently, the most thoroughly investigated and widely utilized serum biomarker for OC diagnosis is cancer antigen 125 (CA125) [9]. Other biomarkers, such as human epididymis protein 4 (HE4), have shown enhanced diagnostic performance compared to CA125 alone [10]. However, the sensitivity and specificity of these methods are insufficient. Additionally, existing imaging techniques fail to adequately address intertumoral or intratumoral heterogeneities and are overly reliant on the subjective judgment of radiologists.

Radiomics, as a rising and dynamic field of research, has recently emerged as a promising solution to address these issues challenges by quantitatively evaluating the characteristics of lesions and extracting potential information through high-throughput analysis of high-dimensional quantitative features collected from multiple medical images [11], particularly in the field of oncology. Furthermore, the integration of radiomics with genomics has led to the development of a novel technique known as radiogenomics. This innovative approach can be utilized to predict or elucidate concealed genetic and molecular attributes, thereby enabling a more comprehensive, precise, and personalized evaluation of tumor biological characteristics. With the rapid advancement and implementation of artificial intelligence (AI) techniques, both radiomics and radiogenomics have significantly evolved in their clinical application for ovarian tumors in recent years, thus facilitating the resolution of more intricate decision-making tasks, such as tumor classification and subtyping, prognosis prediction, disease progression assessment, and identification of abnormal genetic alterations.

Although prior reviews on radiomics and radiogenomics of OC have been conducted [5, 6, 12], the articles included were not sufficiently comprehensive, and the summaries lacked detail. In the initial review of radiomics in OC, Nougaret et al. [6] introduced the role of texture analysis, a method of radiomics in prognostic prediction for OC. Subsequently, Nougaret et al. [12] published another review addressing both radiomics and radiogenomics in OC, but the number of articles included was limited and presented chronologically. In a recent study of this field, Panico et al. [5] focused solely on predicting metastasis, gene mutation, recurrence and chemotherapy response in OC while neglecting to address the application of radiomics for classifying and subtyping benign versus malignant ovarian tumors. Unlike previous research efforts, this review synthesizes the latest literature and organizes our findings based on the role of radiomics and radiogenomics in diagnosing and predicting ovarian tumors, thereby presenting a more coherent framework. For a better understanding of the application of radiomics and radiogenomics, we describe their development and workflow in detail. Furthermore, we provide a thorough summary of their applications in OC to enhance comprehensiveness. Finally, we conclude with an exploration of current challenges alongside potential solutions while offering recommendations aimed at advancing clinical practice as well as discussing future directions in this field.

## Development and workflow of radiomics and radiogenomics

**Fig. 1:**
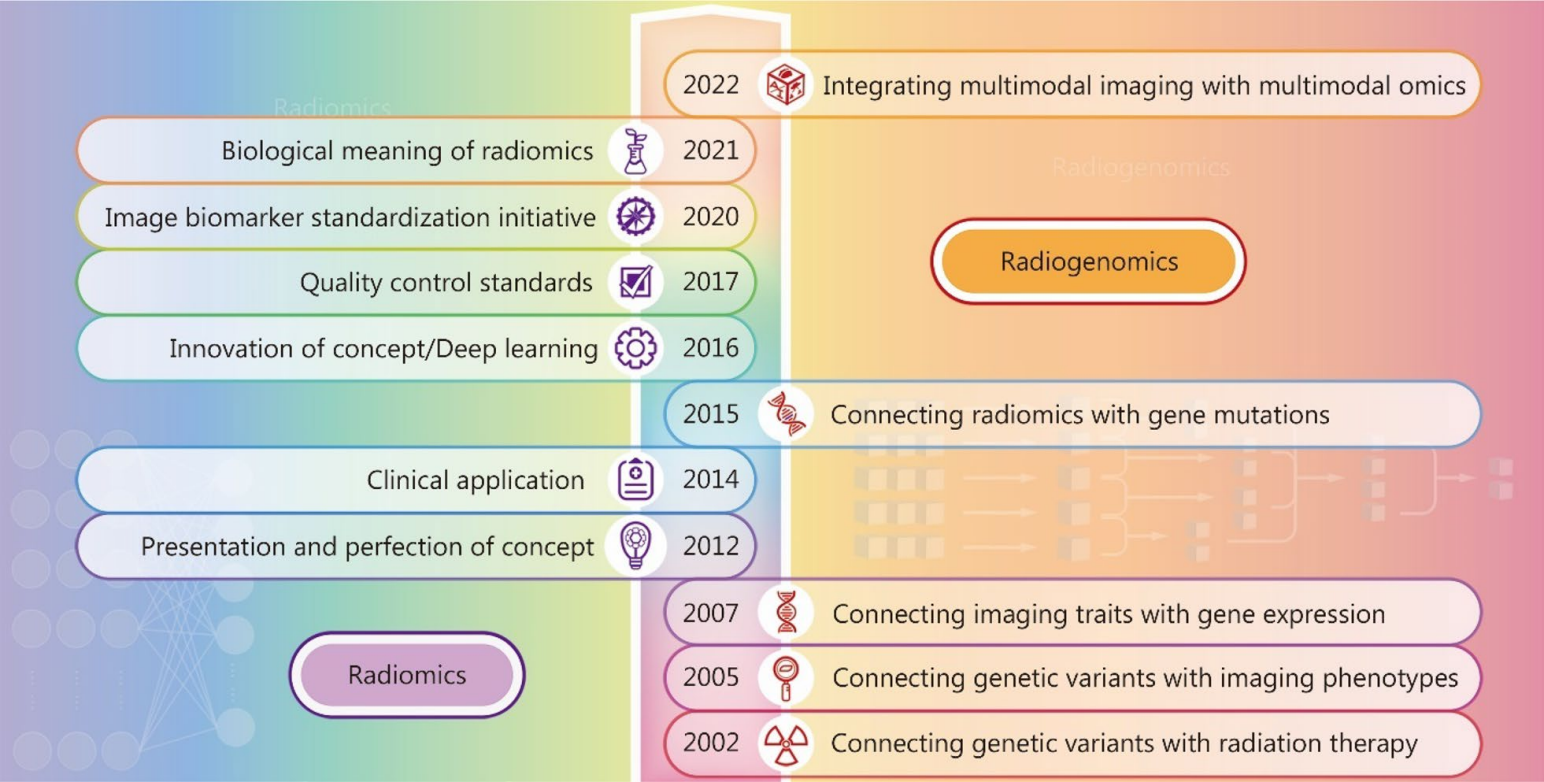
The development and workflow of radiomics and radiogenomics

Radiomics was first introduced by Lambin et al. in 2012 [13], broadly referring to the high-throughput extraction of image features from radiographic images. In the same year, Kumar et al. [14] further elaborated on this concept by emphasizing the high-throughput extraction and analysis of numerous advanced quantitative imaging features derived from various medical imaging images such as CT, PET, or MRI. In 2014, radiomics was applied for the first time in clinical practice [15]. Subsequently, Gillies et al. [16] advanced the concept by incorporating not only imaging features but also clinical and genetic information into quantitative analyses. Furthermore, deep learning (DL) methods were incorporated into radiomics in 2016. In recent years, there has been a growing body of literature focused on standardizing radiomics research while exploring its biological significance [17, 18] (Fig. 1). Radiomics utilizes cutting-edge AI algorithms to transform images into analyzable data, which can be subsequently examined with the aim of enhancing diagnostic and prognostic accuracy while providing personalized treatment options for patients [19]. In this context, machine learning (ML) and DL are both subsets of AI, with DL being a further subset of ML. ML constructs models by transforming medical images into features and subsequently applying algorithms to map these features to corresponding labels, while DL refers to the method that utilizes multi-layer neural networks for learning [20] (Table 1).

**Table 1:** The relationship and difference between machine learning (ML) and deep learning (DL)

Characteristics	ML	DL
Data dependency	It can work with less data	It requires a large amount of data input to achieve good performance
Type of data	Only structured data can be processed	Both structured and unstructured data can be processed
Image segmentation	Manual	Automatic
Feature extraction	Experts are required to perform feature extraction before proceeding further	There is no need to develop a feature extractor for every problem; instead, it tries to extract features from the data on its own
Feature categories	Typically predefined	Allow the creation of new features
Model construction	Models incorporating radiomics features are trained through algorithms	Simultaneously train model when learning features
Approach of operation	The problem is broken down into subparts and a result is produced after solving each part	Take input from a given problem and produce the result. Therefore, it follows an end-to-end approach
Execution time	Requires less time than DL to train models but a long time to test them	Requires a long time to train the model, but less time to test the model
Interpretation of results	Interpretation of the results for a given question is relatively easy	Interpreting the results for a given problem is very difficult because of the cryptic reasoning processes involved
Problem solved	Suitable for solving simple problems	Suitable for solving complex problems
Hardware dependency	Because ML models do not require large amounts of data, they can be run on low-end machines	Large amounts of data are required to work effectively, hence the need for high-end machines
Cost	Low	High

**Fig. 2:**
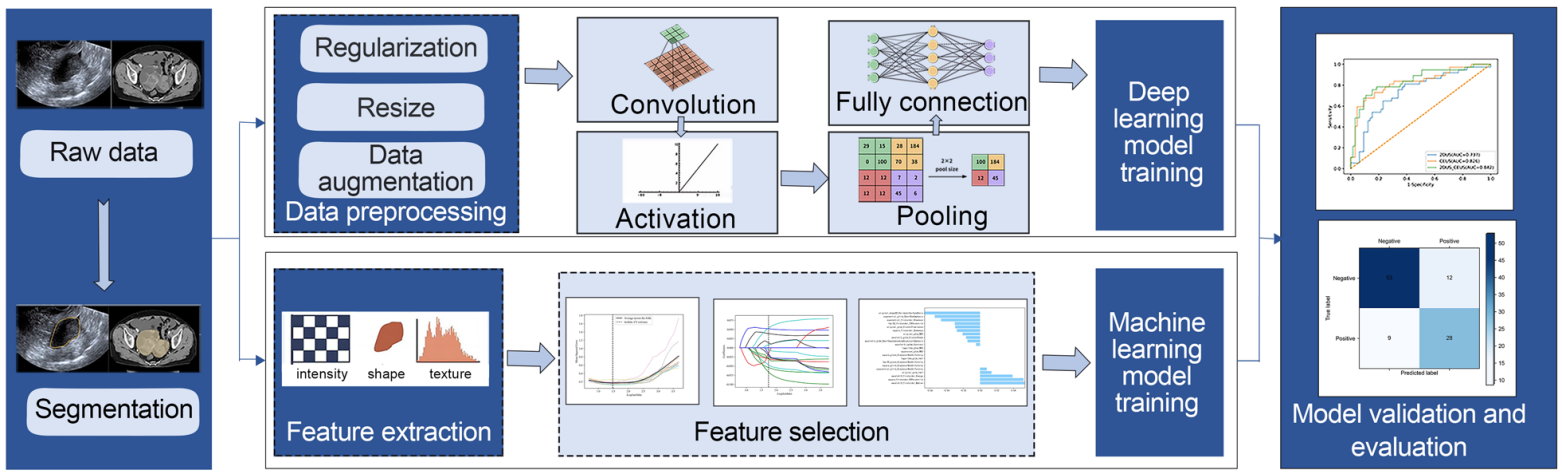
General framework of radiomics. The process for radiomic research applying the machine learning (ML) method includes image acquisition, regions of interest (ROI) segmentation, feature extraction, feature selection, model training, and validation, whereas the deep learning (DL) method includes data preprocessing, convolution, activation, pooling, and full connection

A standard radiomics method typically begins with a radiologist identifying qualitative factors in medical images and culminates in an automated assessment of microscopic structures [21]. This process adheres to a well-established radiomics pipeline, which ML consists of the following steps: (i) image acquisition; (ii) tumor segmentation; (iii) feature extraction; (iv) feature selection; (v) model construction and validation. And convolutional neural network (CNN) as the basic framework of DL, includes the following processes: (i) data preprocessing; (ii) convolution layer; (iii) activation layer; (iv) pooling; (v) fully connected layer (Fig. 2). The first step in radiomics involves the acquisition of standardized and high-quality images [13], and then delineate the regions of interest (ROI) for further analysis. Segmentation can usually be performed in manual, semi-automatic, or fully automatic ways [19]. Unless lesions exhibit complex appearances, manual expert segmentation remains the most effective option [22–25]. From the ROIs, various types of radiomic features can be extracted. These features are primarily categorized into two main types: manually defined features and DL-based features, which are outputs generated by algorithms utilizing a stacked neural network structure [26]. The former can further be classified as shape features, texture features, and intensity features [27–29]. Due to the large number and complexity of extracted features, overfitting and low generalization may occur; thus, it is necessary to filter out the most meaningful attributes. Commonly employed techniques include least absolute shrinkage and selection operator (LASSO) regression, recursive feature elimination (RFE), principal component analysis (PCA), and independent component analysis (ICA) [11]. The final step and ultimate goal of the radiomics process is model construction. Consequently, validating model performance is crucial to demonstrate its potential for clinical application.

Radiogenomics was initially proposed to investigate the relationship between genetic factors and radiation therapy [30]. Subsequently, it evolved to encompass radiomics and gene expression analysis [31]. The current definition of imaging genomics now integrates multi-omics data generated by next-generation sequencing (NGS) with multimodal imaging data obtained during clinical practice [32] (Fig. 1). Radiogenomics involves three key steps: (i) extraction of imaging feature; (ii) extraction of multi-omics feature; and (iii) association or integration of imaging and multi-omics data. Imaging features are extracted from radiological and histopathological images using methodologies similar to those employed in radiomics. Conversely, multi-omics features are derived from various biological sources, including genes, transcriptomes, and proteins, among others. The fusion of imaging and multi-omics data necessitates the application of advanced analytical methods such as canonical correlation analysis (CCA), which can uncover hidden associations between the two datasets. Additionally, integration-based models are employed to combine these datasets effectively for predicting patient outcomes [33]. With algorithmic advancements and the increasing accessibility of acquiring and exchanging genetic material, radiogenomics is poised to reach its full potential.

## Clinical applications of radiomics and radiogenomics in OC

**Fig. 3:**
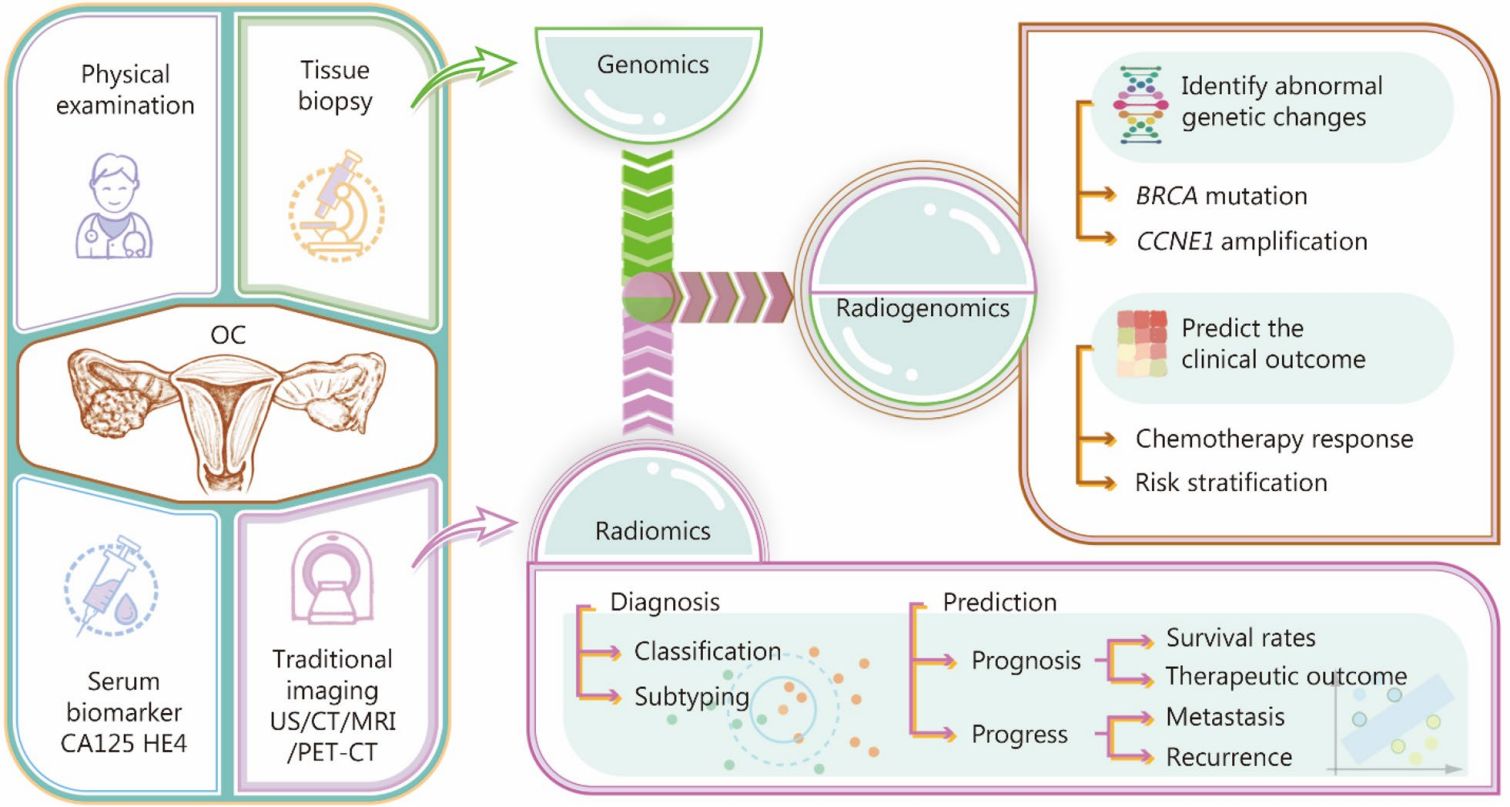
The application of radiomics and radiogenomics in ovarian cancer (OC). The application of radiomics involves the diagnosis and prediction of OC, while the application of radiogenomics involves identifying abnormal genetic changes and predicting clinical outcomes in OC patients. CA125 cancer antigen 125, CT computed tomography, HE4 human epididymis protein 4, PET/CT positron emission tomography/CT, US ultrasound

Radiomics and radiogenomics provide a diverse array of potential applications in the field of oncology, offering objective and quantitative methodologies for evaluating tumor phenotypes and predicting clinical outcomes. This review will explore the prospective applications of radiomics and radiogenomics in OC. For radiomics, two main applications are summarized: the diagnosis and prediction field of OC, which involves both the classification and subtyping of OC, as well as forecasting disease prognosis and progression which the former includes survival rates and treatment outcome, while the latter addresses metastasis and recurrence. In terms of radiogenomics, two key implications are highlighted: detecting abnormal changes in known genes, along predicting clinical outcomes by integrating genetic factors with imaging features (Fig. 3).

### Radiomics-based diagnosis of OC

#### Classification of OC

Precise classification of ovarian masses is essential for the development of optimal treatment strategies that preserve fertility in benign lesions and improve prognosis for patients with ovarian malignancies. Benign masses may be managed conservatively or removed through minimally invasive surgical techniques [34], while patients with suspected malignant masses should be transferred to a tertiary care facility specializing in OC cases [35]. Given the morphological overlap between benign and malignant ovarian masses, traditional imaging techniques often face challenges in accurately identifying malignant tumors and providing appropriate treatment, resulting in poor prognosis of OC patients [36, 37]. Therefore, numerous studies have investigated the application of radiomics methods for classifying ovarian tumors.

In 2013, Faschingbauer et al. [38] extracted texture features from ultrasound images of 105 ovarian lesions, classified the tumors using a support vector machine (SVM) approach and compared their performance with the subjective evaluations of examiners possessing varying levels of ultrasonography experience. The texture analysis demonstrated significantly superior diagnostic performance compared to physicians with low or medium levels of experience. Subsequently, Aramendía-Vidaurreta et al. [39] developed a DL model to differentiate between benign and malignant ovarian tumors based on ultrasound images, achieving satisfactory results with an area under the receiver operating characteristic (ROC) curve (AUC) of 0.997. In contrast to the studies that used a limited number of radiomics features, Zhang et al. [40] obtained 1714 features from 4 MRI protocols for each lesion and constructed a more robust model yielding an AUC of 0.975 in the cross-validation cohort. To assess the accuracy in classifying benign and malignant ovarian tumors using contrast-enhanced (CE)-CT radiomics analysis based on ML, Li et al. [41] developed 2 models, a radiomics model and a mixed model incorporating 3 clinical predictors of HE-4, ascites, and margin, demonstrating strong performance in the validation cohort with AUCs of 0.91 and 0.96 respectively. These studies collectively illustrate that AI-based imaging methods exhibit robust diagnostic capabilities in distinguishing between benign and malignant ovarian tumors.

In recent years, borderline ovarian tumors (BOTs) have been recognized as distinct lesions from benign and malignant ovarian tumors due to their diverse morphological characteristics [42]. Therefore, some researchers have focused on differentiating BOTs from benign or malignant ovarian tumors. Liu et al. [43] created 4 models, two-dimensional (2D)/three-dimensional (3D) sagittal fat-suppressed (FS) T2-weighted imaging (T2WI), and 2D/3D coronal T2WI models, to distinguish between BOTs and cancers, concluding that the 3D sagittal T2WI model exhibited superior diagnostic ability with an AUC of 1.000 in the testing group. This study represents a pioneering effort to investigate the classification of ovarian tumors using MRI-based radiomics features through both 2D and 3D segmentation methods. The 3D model, which encompasses information from the entire lesion, provides a more accurate representation of tumor heterogeneity compared with its 2D counterpart. However, this model is limited by its predominantly focus on singular elements, thereby neglecting the impact of clinical indicators and traditional imaging features on diagnosis. Wei et al. [44] utilized FS-T2WI for modeling, providing additional evidence for the use of T2WI-based radiomics in BOTs. Their research indicated that a combined model integrating radiomics features with clinical data and conventional radiological characteristics could enhance diagnostic performance and generalizability (AUC = 0.86). Furthermore, their study included patients from multiple facilities and conducted both internal and external validation, thereby strengthening the robustness of their findings. However, it is important to note that this model exclusively incorporates FS-T2WI, which differs from the typical clinical reading scenario that generally includes T1-weighted imaging (T1WI), T2WI, and diffusion-weighted imaging (DWI). These studies have demonstrated significant advancements in utilizing MRI-based radiomics for the diagnosis of ovarian tumors. In the study conducted by Qi et al. [45], 3 types of ovarian neoplasms (benign, borderline, and malignant tumors) were categorized into two subgroups: benign versus borderline/malignant (task 1) and borderline versus malignant (task 2). Additionally, they compared the efficacy of various techniques in identifying these subgroups, including evaluations by senior sonographer, junior sonographer, radiomics model analysis, clinical model analysis, and a combined approach. Their findings indicated that the radiomics model performed satisfactorily when compared to assessments made by senior sonographers in both tasks (AUC = 0.79 for senior sonographers and 0.88 for radiomics models in task 1; AUC = 0.61 for senior sonographers and 0.84 for the radiomics model in task 2). Furthermore, a nomogram comprising CA125 levels, lesion location, ascites presence, and radiomics signatures yielded optimal performance (AUC = 0.91 in task 1; AUC = 0.89 in task 2). The research is based in the ultrasound, currently regarded as the first-line imaging mode for diagnosing ovarian tumors, thereby demonstrating substantial clinical practicability.

The accumulated evidence from the studies indicates that radiomics analysis holds significant potential as a non-invasive diagnostic tool for OC. Specifically, it can enhance both accuracy and objectivity, thereby serving as an effective decision-making instrument to facilitate individualized treatment. However, most studies primarily categorize ovarian tumors into benign and malignant classifications, often including borderline tumors within these categories. BOTs exhibit pathological characteristics and biological behavior that lie between benign and malignant tumors. Accurate identification of BOT is essential for determining appropriate treatment strategies. Consequently, there is an urgent need for further exploration of radiomics methodologies aimed at simultaneously distinguishing among these three tumor types in future research endeavors.

#### Subtyping of OC

Neoadjuvant chemotherapy (NAC) may be considered for the treatment of Federation of International of Gynecologists and Obstetricians (FIGO) stage III or IV epithelial ovarian cancer (EOC), particularly HGSOC when complete debulking is unlikely to be achieved through primary cytoreductive surgery. Conversely, in cases of carcinoma resistance to conventional taxane or platinum-based chemotherapy, such as ovarian clear cell carcinoma (OCCC), primary cytoreductive surgery is recommended [35]. In clinical practice, histological diagnosis is typically established via surgery intervention or tissue biopsy; however, this approach may result in misdiagnosis due to intratumoral heterogeneity. Even though intraoperative frozen sections facilitate histological classification, the invasive nature of this procedure and the associated increase in intraoperative duration adversely affect patient prognosis [46]. According to the literature listed in this review, radiomics has demonstrated superior accuracy in tumor evaluation compared to traditional imaging semantic indicators for subtyping of OC.

Previous studies have supported a dualistic model of carcinogenesis that categorizes EOC into two distinct types based on clinicopathological and molecular characteristics [47, 48]. Type I EOC encompass LGSOC, endometrioid carcinomas, OCCC, mucinous carcinomas, and malignant Brenner tumors, while type II EOC include HGSOC, carcinosarcomas, and undifferentiated carcinomas [49]. Type I EOC typically exhibit an indolent clinical course with limited progression; ipsilateral oophorectomy may be beneficial in the early stages, but most metastatic type I tumors are chemoresistant. In contrast, type II cancers are comparatively aggressive and are usually diagnosed at advanced stages requiring more invasive surgical interventions; however, standard platinum-based chemotherapy is generally effective in most cases [50]. Therefore, some articles emphasize differentiating type I and type II EOC subtypes prior to therapy to facilitate improved management strategy selection and prognostic evaluation. Qian et al. [51] demonstrated that through MRI radiomics analysis, a mixed model incorporating combined radiomics features performed comparably to the conventional model (AUC = 0.97 for the mixed model versus AUC = 0.96 for the conventional model). Although not statistically significant, the radiomics model showed potential utility. However, their study is limited by the absence of a validation group. Subsequently, Jian et al. [52] extracted radiomics features from multiparametric MRI, including FS-T2WI, DWI, apparent diffusion coefficient (ADC) maps, and contrast-enhanced T1-weighted imaging (CE-T1WI), developing a composite model that demonstrated strong performance with an AUC of 0.85 during external validation. The study was externally validated and collected patient data from multiple centers to enhance the reliability of the findings. However, incorporating clinical data may further improve the model. To explore the potential of using radiomics signatures based on DWI and ADC maps for identifying EOC, Xu et al. [53] extracted 390 features to establish the radiomics model. They subsequently created a combined model by integrating these radiomics features with clinical characteristics. The results revealed that the radiomics model outperformed the clinical model in diagnosing early-stage type I and type II EOC (AUC = 0.91 vs. 0.74). The diagnostic accuracy of the nomogram was equivalent to that of the radiomics model, yielding a networked readiness index (NRI) value of  -0.1591. Yao et al. [54], for instance, were among the first to apply ultrasound radiomics for subtyping type I and type II EOC and constructed a nomogram exhibiting superior performance compared to both the clinical and radiomics models (AUC = 0.82 vs. 0.73 vs. 0.74). Similarly employing the ultrasound method as their foundation, Tang et al. [55] utilized 6 ML methods to identify an optimal approach before constructing a nomogram combining radiomics and clinical factors which displayed robust performance with an AUC of 0.89 in the validation set. In a recent study conducted by Li et al. [56], CE-CT was used for subtyping EOC, during which they compared 6 modeling methods and ultimately selected logistic regression method. Then, they integrated clinical factors to develop a combined model that achieved an AUC of 0.93, surpassing the performance of individual models.

There are various subtyping approaches for OC aimed at achieving more effective subtype-specific treatments. Research has indicated that HGSOCs typically present with late peritoneal carcinomatosis; however, they respond more favorably to NAC compared to non-HGSOCs [57, 58]. In advanced HGSOC, NAC is particularly advantageous due to its chemosensitivity, whereas non-HGSOCs tend to benefit more from primary cytoreductive surgery. Therefore, some studies have focused on distinguishing between HGSOC and non-HGSOC subtypes. For example, one study integrated clinical characteristics and textural features from CE-CT to develop a successful combination model [59]. Another study by Wang et al. [60] utilized features across multiple categories to create a comprehensive radiomics model based on a large sample size. Although the data were collected from multiple sites, this approach overlooked clinical features and thus presented an opportunity for improvement. In a separate investigation conducted by Li et al. [61], 138 patients with SOC confirmed via histology were retrospectively assessed to evaluate the efficacy of MRI radiomics in distinguishing between HGSOC and LGSOC. Compared to either the clinical or radiomics models, a novel combination model had greater diagnostic effectiveness with an AUC of 0.93. Given the significant differences in therapy schedules for EOC and non-epithelial ovarian cancer (NEOC), Zhu et al. [62] extracted preoperative CT images from 101 patients with pathologically confirmed OC to evaluate the feasibility of radiomics to distinguish between EOC and NEOC using the logistic regression analysis. A nomogram developed by integrating clinical and radiomics signatures (AUC = 0.87) outperformed the radiomics (AUC = 0.78) and clinical models (AUC = 0.81), respectively. Within the range of NEOC, sex cord-stromal tumor (SCST) typically present as solid masses; however, diagnostic challenges may frequently arise if necrosis, bleeding, edema, or cystic degeneration is observed. Recently, Cheng et al. [63] conducted a pioneering radiomics study to differentiate between SCST and EOC by constructing a robust mixture model that effectively distinguishes between these two entities with an AUC of 0.88. OCCC represents a distinct subtype of EOC where debulking surgery is recommended as the primary treatment, while conventional chemotherapy has shown limited efficacy. Therefore, early and accurate identification of the OCCC subtype before treatment is crucial to avoid unnecessary NAC. An integrated model combining clinical characteristics with radiomics features collected from CE-CT has been established to detect OCCC alongside other types of EOC, yielding an AUC of 0.86. This study revealed that radiologists employing this model might improve subtyping sensitivity [64]. Although various subtyping methods exist, the ultimate goal remains optimizing patient treatment while improving prognosis outcomes. The aforementioned studies suggest that radiomics holds substantial potential in OC subtyping and enhanced resolution can be achieved through the amalgamation of clinical characteristics and traditional imaging parameters.

At present, the majority of studies related to OC subtyping are primarily focused on distinguishing between type I and type II EOC, as well as EOC versus NEOC, and HGSOC versus non-HGSOC, with only a limited number of investigations dedicated to identifying OCCC. However, numerous other OC subtypes exhibit distinct biological differences that correlate with varying degrees of malignancy and drug sensitivity. Therefore, the identification of these additional OC types is of significant clinical importance and warrants further exploration by researchers.

### Radiomics-based prediction of OC

#### Predict the prognosis

*Survival rates*. Clinical outcomes can vary significantly, even among epithelial ovarian cancer (EOC) patients who have been treated homogeneously and present with the same tumor stage, due to the inherent heterogeneity of the tumor. Identifying patients at high risk for mortality prior to surgery may facilitate decisions regarding the necessity for more aggressive therapies and stringent monitoring. However, there remains a lack of reliable prognostic and predictive biomarkers [65]. Consequently, it is imperative to develop a prognostic prediction tool that supports clinical judgment and enables personalized precision therapy for OC patients.

Based on MRI findings, Zhang et al. [40] established a survival analysis model for OC patients. The Kaplan–Meier method was utilized to obtain, analyze, and model the MRI radiomics characteristics that were most closely associated with the survival status of patients using LASSO regression. The results demonstrated that this radiomics model could offer a high-precision assessment of survival for OC patients. To create and validate a radiomic-clinical nomogram for assessing overall survival (OS) in SOC patients after surgery, Hong et al. [66] conducted a study to develop a radiomics signature using LASSO regression on a training set comprising 1301 radiomics characteristics extracted from OC lesions in CT images. Then, they constructed a radiomic-clinical nomogram through multivariate Cox regression analysis by integrating the radiomics signature with clinical variables. The findings revealed that the nomogram included the radiomics signature along with 4 clinical predictors: age, tumor size, pathological stage and tumor grade. This nomogram exhibited favorable discrimination in both the training and validation sets. In a retrospective analysis involving 734 HGSOC patients, Zheng et al. [67] created a DL model based on Vision Transformer (Vit) to predict OS using preoperative CT images, leading to promising results in predicting survival with an AUC of 0.822 in the training cohort and 0.823 in the validation cohort. Lu et al. [68] collected 657 quantitative mathematical descriptors from preoperative CT scans of 364 patients with EOC to develop a novel mathematical descriptor for prognostic and molecular phenotypes defined by radiomics, possessing predictive value. Utilizing the LASSO method, they identified 4 weighted features associated with OS, which were employed to calculate a radiomic prognostic vector (RPV) score for each tumor. Patients were subsequently categorized into low-risk, medium-risk, and high-risk subgroups based on their RPV scores using an unsupervised K-means clustering technique. Significant differences in OS were observed among patient groups according to RPV stratification in both discovery dataset and two independent validation datasets. In a separate study, this radiomics biomarker was evaluated in the Department of Gynecologic Oncology at Kliniken Essen-Mitte (KEM), an OC center of excellence accredited by the European Society of Gynecological Oncology (ESGO), to assess its applicability. It was found that patients with lower RPV exhibited improved progression-free survival (PFS) compared to those with high RPV. Furthermore, in a multivariable Cox regression model, RPV showed a significant effect on PFS [69]. Despite these promising findings, external verification and further testing through multicenter studies are required to establish its suitability for clinical application.

Risk stratification of patients prior to surgery enables more personalized treatment approaches. For those with poor long-term survival, alternative pathways can be recommended, such as NAC, and the early introduction of novel targeted therapies. Several previous studies have endeavored to develop prognostic prediction tools based on molecular profiles obtained from tumor biopsies [70, 71]. However, the translation of these molecular prognostic models into routine clinical practice remains challenging due to several factors: significant intratumoral heterogeneity leading to limited prognostic power, the invasive nature of biopsy procedures, high testing costs, and crucially, the considerable time constraints associated with molecular testing protocols. In contrast, radiomics leverages information extracted from patients’ routine preoperative imaging scans at the time of disease presentation. This approach is readily accessible without additional costs or time delays and has demonstrated substantial predictive capability. Nevertheless, most current research in this field is predominantly focused on CT and MRI modalities; thus, it is imperative to explore radiomics models derived from other imaging techniques such as ultrasound and CE-CT to identify optimal prognostic models.

*Therapeutic outcome.* The application of radiomics in predicting therapeutic outcomes is primarily focused on forecasting responses to residual disease and chemotherapy, as these are the two most critical factors influencing treatment strategies. For patients with HGSOC, the standard treatment course remains primary debulking surgery (PDS) followed by platinum-based chemotherapy. The presence of residual disease at the time of PDS serves as a significant prognostic indicator [72–74]. According to reliable evidence, individuals with advanced HGSOC after PDS may achieve optimal prognosis through complete resection of all visible tumors (R0 resection) [72, 73]. However, PDS does not confer any survival benefit for patients with a low probability of attaining R0 resection, but it can significantly increase perioperative morbidity [75]. Consequently, the recommended approach for these patients is interval tumor reduction surgery (IDS) followed by NAC [76–78]. It is essential to identify patients who are unlikely to achieve R0 resection before surgery. Preoperative abdominal CT imaging and laparoscopy-based minimally invasive techniques are frequently used clinical tools for predicting residual disease at PDS. Nevertheless, the laparoscopic score may be limited by its invasiveness, cost considerations, and potential risk for disease metastasis. Similarly, non-invasive methods based on CT depend heavily on subjective assessments from radiologists [79, 80]. Currently, radiomics has emerged as a non-invasive and objective method that can effectively predict residual disease as delineated in this paper [81, 82].

To determine the relationship between radiomics features, either independently or in conjunction with clinical data, and residual disease at the time of surgery, Rizzo et al. [83] extracted 516 radiomics features from ovarian masses using a 3D ROI derived from CT images of HGSOC patients. They employed the Chi-square test to evaluate the association between representative cluster radiomics features and residual disease. The results demonstrated that mass size, randomness, and homogeneity were significantly correlated with residual disease. To develop a radiomic-clinical nomogram based on preoperative MRI for patients with advanced HGSOC that can predict residual disease, Li et al. [81] analyzed a cohort of 217 individuals with advanced HGSOC. To ensure consistency in radiomic feature extraction between patients presenting bilateral cancers and those with unilateral tumors, two fusion methods were implemented: maximal volume of interest (MV) and maximal feature value (MF). Radiomics signatures were generated by combining the LASSO classifier alongside the minimum redundancy maximum relevance (mRMR) technique. A radiomic-clinical nomogram was constructed incorporating both traditional clinicoradiological parameters and these novel radiomics signatures through multivariate logistic regression analysis, followed by performance evaluation using a validation set. The findings revealed that this nomogram achieved an AUC of 0.80 in the validation set, cohort, demonstrating superior predictive capability compared to traditional clinicoradiological metrics as well as MF-based radiomics signatures, which yielded AUCs of 0.62 and 0.74 respectively. Additionally, another study developed a nomogram utilizing MRI data for predicting residual disease. This investigation quantified metastases in abdominal and pelvic regions as independent factors influencing R0 resection inclusion within the nomogram framework, achieving an impressive AUC of 0.90 [82].

When immediate primary surgery is not feasible, NAC is recommended prior to delayed primary surgery (DPS) for patients with advanced HGSOC. For those who exhibit resistance to chemotherapy, early surgery intervention or targeted immunotherapy may be considered [74]. The most reliable method for assessing the response to chemotherapy is the chemotherapy response score, which requires omental surgery and is not widely accessible. Therefore, there is a pressing need for an alternative approach that offers both high accuracy and ease of use [84]. With the advent of radiomics, non-invasive prediction of chemotherapy remission and platinum resistance has become achievable, demonstrating commendable performance. Rundo et al. [85] explored whether omental tumor volume and CE-CT-based radiomics could effectively predict the complete response to NAC in HGSOC patients. The results showed that pre- and post-NAC measurements of omental tumor volume, along with a larger percentage change in response to NAC, were significantly correlated with achieving a complete response. Furthermore, the model incorporating radiomics features obtained a higher AUC in the training set. While the volumetric model had the highest AUC in the validation set, integrating radiomics data significantly enhanced the negative predictive value (NPV) from 0.61 to 0.79, thereby indicating improved reliability in identifying non-responders at earlier time points. In a recent study by Li et al. [86], recurrence within 6 months following the completion of platinum-based chemotherapy was defined as platinum resistance. They developed a radiomics nomogram that combined radiomics signatures with 3 clinical characteristics. This nomogram demonstrated superior performance compared with the clinical mode, achieving a higher AUC of 0.80 vs. 0.75. Platinum-based chemotherapy remains the standard treatment before DPS; however, patients with platinum resistance should be considered for alternative non-platinum therapies. The establishment of this model is anticipated to improve long-term prognostic outcomes for advanced HGSOC patients [87, 88].

Surgery and chemotherapy represent the fundamental treatments for OC. The aforementioned studies have demonstrated that radiomics can effectively predict residual disease and chemotherapy sensitivity in patients with OC. With ongoing research into OC treatment, additional modalities such as targeted therapy and immunotherapy are gradually offering hope to patients with inoperable or chemotherapy-resistant OC. To alleviate the financial and physical burdens associated with these treatments, further predictions regarding their therapeutic efficacy will be essential in future endeavors.

#### Predict the progress

*Metastasis.* According to previous studies, the 5-year overall survival rate for early-stage disease is approximately 92%, while the rate for late-stage disease is only 29%, suggesting that advanced metastasis significantly impacts patient survival [89]. Early detection of metastasis is crucial for accurate staging of OC and has the potential to enhance prognosis, treatment options, and overall survival rates for patients [90]. However, research indicates that screening methods using CA125, ultrasound, and other technologies have proven ineffective in reducing mortality associated with OC, and no effective screening techniques have been developed [91]. In recent years, the application of radiomics in OC has gradually evolved to predict metastasis with high sensitivity and specificity. This advancement holds promise for further guiding clinical management. Ai et al. [92] recruited 101 patients with pathologically confirmed metastases to investigate if radiomics features alone or in combination with clinical indicators could reliably predict the status of metastases. The results demonstrated that the combined model outperformed both radiomics and clinical models in predicting metastasis (AUC = 0.86 vs. 0.83 vs. 0.82). Notably, up to 75% of individuals diagnosed with stage III–IV disease exhibit lymph node metastases, while approximately 25% of patients with stage I–II disease also present lymph node involvement among those affected by HGSOC [93, 94]. The FIGO staging system for EOC is significantly influenced by lymph node status [95–97]. For instance, patients identified as having stage I lymph node metastases may be reclassified as stage III or IV [98, 99], highlighting the substantial impact of lymph node status on the EOC staging. According to guidelines established by the American College of Radiology and the European Society of Urogenital Radiology (ESUR), CT is currently regarded as the first-line imaging method for OC staging and follow-up [100]. However, with a sensitivity ranging from 48 to 80%, its predictive capability for lymph node metastasis remains insufficient [101]. Therefore, it is imperative to explore non-invasive techniques for preoperatively predicting lymph node metastasis to enhance clinical decision-making. In a study by Chen et al. [102], a total of 256 patients with pathologically confirmed lymphatic metastasis were included. The results indicated that the radiomics approach performed better than the CT lymph node report (AUC = 0.75 vs. 0.72), and when combined, these methods yielded a composite model exhibiting superior predictive performance (AUC = 0.84).

Clinically, the identification of small peritoneal metastasis (PM) in biopsies leads to approximately 30% of early-stage OC patients being upstaged postoperatively [103]. Early detection of peritoneal tumor seeding is essential for clinicians to improve preoperative staging accuracy and guide personalized therapy for OC, as it significantly influences therapeutic strategies and outcomes. The most commonly utilized imaging modalities for assessing PM are CT and MRI; however, they exhibit limitations in detecting micronodular peritoneal seeding when tumor masses are absent [104, 105]. DWI may offer a more precise evaluation of PM status in OC, although its effectiveness largely depends on the expertise of physicians. Therefore, developing an impartial tool for the precise identification of PM is essential. According to two recent investigations, the MRI-based radiomics nomogram demonstrated exceptional performance in recognizing PM status among patients with EOC [106, 107]. Despite these findings being encouraging, the small sample sizes employed in these studies may lead to bias in their results. Additionally, both investigations were conducted at a single center and lacked external data for validation, which limits the generalizability of their conclusions. The latest study addressed these limitations. Wei et al. [108] constructed clinical models alongside radiomics and DL models and subsequently integrated them into an ensemble model that exhibited superior predictive performance. This study also compared the predictive capabilities of this model against those of radiologists, revealing that the former outperformed human assessments while confirming that the diagnostic performance improved among various experienced radiologists following model-assisted interpretation.

The studies mentioned above were conducted to predict the metastasis of the peritoneum and lymph nodes, which are the most common metastatic sites of OC. The National Comprehensive Cancer Network (NCCN) guidelines recommend selective resection of all peritoneal surfaces and any metastatic lymph nodes suspected of harboring cancer, as this approach aims to enhance survival rates. Currently, the diagnosis of metastatic lesions primarily relies on laparoscopy, a method that is both invasive and costly [109]. Radiomics, as a non-invasive technique, addresses these challenges and has demonstrated superiority over traditional imaging modalities in predicting metastasis. However, its reliability necessitates further investigation through multi-center trials and prospective studies.

*Recurrence.* HGSOC is the most prevalent and aggressive subtype of OC, characterized by a high rate of tumor recurrence that contributes to patient mortality [110, 111]. Despite this, there are currently no reliable predictive biomarkers for clinical application [112]. Recent literature suggests that radiomics can enhance the predictive value of clinical indicators by extracting quantitative features in a high-throughput manner to predict tumor recurrence. Wang et al. [113] used DL to develop a noninvasive model for predicting recurrence in patients with HGSOC based on CE-CT images. The results showed that the C-index of the model was 0.713 and 0.694 for the two validation cohorts, respectively. In another study also based on CE-CT images, Chen et al. [114] revealed that the addition of radiomics features significantly improved the predictive ability of clinical models for recurrence, which combined model achieved an AUC of 0.77. As the inaugural study to utilize β-2-[^18^F]-fluoro-2-deoxy-D-glucose (^18^F-FDG) PET/CT, Wang et al. [115] sought to determine whether radiomics features extracted from the PET and CT components of ^18^F-FDG PET/CT images, in conjunction with clinical characteristics and PET metabolic parameters, could predict PFS in HGSOC patients. The study found that the clinical + PET model performed better in terms of prognostic performance than other models. Previous studies have shown that MRI-based radiomics is also effective for predicting the recurrence of OC [116–118]. Among them, Wang et al. [118] showed that the radiomics model based on T2WI had the best prediction ability by modeling and comparing different sequences of MRI. Moreover, Li et al. [116] adopted the DL method and conducted external verification, which has broader applicability and credibility. Yao et al. [119] first built a radiomics model based on the ultrasound and integrated it with clinical characteristics to create a promising model, with an AUC of 0.83, for predicting the recurrence of OC.

Preoperative identification of recurrence in patients with OC is crucial, as it can inform personalized treatment and monitoring strategies, including the selection of chemotherapy agents. Current research indicates that clinical features such as CA125 and FIGO stage are associated with OC recurrence [120]. However, traditional clinical biomarkers provide limited insights into tumor characteristics and often require invasive procedures. The aforementioned studies have established AI models for predicting recurrence based on multiple imaging modalities, all yielding significant findings. This underscores the effective application of radiomics in recurrence prediction, which is vital for monitoring disease progression. Nevertheless, current predictions primarily focus on HGSOC or EOC, both of which exhibit relatively high recurrence rates. In contrast, germ cell tumors, sex cord-stromal tumors, and metastatic OC also remain at risk for recurrence following therapy. Consequently, predicting the recurrence across a broader range of OC subtypes is essential for improving the overall prognosis of OC patients.

### Application of radiogenomics in OC

#### Identify abnormal genetic changes

*BRCA1/2* mutations in both germline and somatic cells represent the most prevalent mechanism driving homologous recombination deficiency, which is observed in approximately 50% of EOC and results in DNA repair deficiencies through homologous recombination [121]. Several studies have indicated that patients with *BRCA*-mutant HGSOC tend to exhibit a higher likelihood of survival compared to those with *BRCA* wild-type HGSOC [122–124]. A more favorable prognosis for *BRCA*-mutant HGSOC has been associated with increased sensitivity to platinum-based chemotherapy and distinct tumor biology, providing survival benefits independent of chemotherapy sensitivity [123, 125]. Additionally, poly ADP-ribose polymerase (PARP) inhibitor therapies may serve as beneficial adjuncts to maintenance treatment for *BRCA*-mutant HGSOC during this interim period [126]. Therefore, detecting the status of *BRCA* mutation is crucial for personalized treatment stratification. Genetic testing currently serves as the primary method for clinical assessment of *BRCA* gene mutations; however, it can be costly and time-consuming, and a single sample often fails to encompass the entire tumor [127]. Consequently, there is an urgent need to develop imaging models capable of non-invasively and comprehensively evaluating *BRCA* mutations in OC. There are conflicting findings regarding the ability of radiomics features to predict *BRCA* mutational status. Meier et al. [128] investigated the relationship between *BRCA* mutational status and inter-site heterogeneity but found no significant evidence linking them. In another study conducted by Li et al. [129], three models were developed using 2D and 3D CE-CT radiomics characteristics to predict *BRCA* mutational status. However, Delong tests revealed no substantial differences in performance among these models. In a multicenter study, authors constructed both radiomics and DL models to predict *BRCA* mutation and found that only the model incorporating clinical factors achieved an AUC of 0.75, while all other models exhibited poor predictive performance [130].

Amplification of the cyclin E1 gene (*CCNE1*) has frequently been associated with chemoresistance and primary treatment failure in HGSOC patients, as well as poor survival outcomes [131–133]. In vitro studies have shown that tumors exhibiting increased *CCNE1* levels are susceptible to inhibition by cyclin-dependent kinase 2 (CDK2) or proteasome inhibitors [134, 135]. Consequently, the amplification status of *CCNE1* may aid in stratifying individuals with HGSC for targeted therapies. In a study examining tumor heterogeneity, gray-level correlation matrix-based texture features from all sites suspected of HGSOC involvement on preoperative CT were calculated and divided into 5 clusters using a Gaussian mixture model. The intersite similarity matrix (ISM) was generated by calculating the similarity between site pairings. Based on ISM, the textural heterogeneity index between sites was computed and compared to *CCNE1* amplification status. The result showed that *CCNE1* amplification predominately occurred in individuals exhibiting greater variability in inter-site texture metrics [136].

The application of targeted therapies and the prognosis of carcinomas are intricately linked to abnormal gene expression. The emergence of radiogenomics addresses the limitations associated with time-consuming and costly genetic testing, demonstrating its utility in predicting *BRCA* mutations and *CCNE1* amplification. Looking forward, the prediction of additional existing targets, such as *TP53*, will increasingly depend on advancements in radiogenomics.

#### Predict the clinical outcome

Nowadays, the most common treatment approach for advanced HGSOC is NAC followed by DPS [127]. However, 39% of patients do not exhibit any discernible improvement following neoadjuvant therapy with paclitaxel and carboplatin [137]. If it were possible to identify potential non-responders who would benefit from urgent primary surgery before the initiation of therapy, patient care would be significantly enhanced. Individual data streams, including clinical characteristics [138], CA125 levels [139], CT imaging [127], and circulating tumor DNA (ctDNA) [140], have been the focus of prediction studies. The improved predictive potential capability of integrative models for complex endpoints has been successfully shown in numerous cancer types [141, 142]. Therefore, Crispin-Ortuzar et al. [143] developed an integrative radiogenomic framework to predict chemotherapy responses. In the correlation study exploring disease burden, the results indicated that both CA125 levels and the *TP53* mutant allele fraction (MAF) accessed via ctDNA significantly correlated with total disease burden at baseline [total volume, number of lesions, and summed response evaluation criteria in solid tumors (RECIST 1.1) diameters] and showed a significant positive correlation with the summed RECIST 1.1 diameters post-chemotherapy. Then, the authors trained four models by successively adding clinical and molecular features: (i) age, FIGO stage, and treatment; (ii) CA125; (iii) radiomics features; and (iv) ctDNA to predict RECIST 1.1. The results showed in an independent external cohort that the full model achieved an AUC of 0.80, similar to the radiomics model (0.78), compared to 0.47 and 0.50, respectively for the clinical and CA125 models [143]. The integrative radiogenomics framework showed good predictive power. In another study focused on predicting platinum resistance in OC, the predictive performance of integrated models combining genomic data such as human single-nucleotide polymorphisms (SNPs) related to human sulfatase 1 (*SULF1*) and CT radiomics features derived from pretreatment CT images was superior to that of *SULF1* model combined with SNPS alone or using the radiomics model independently [144]. Furthermore, a multimodal data model proved effective for risk stratification among patients. In a study aiming at predicting patient mortality, a multimodal model that includes histopathological, radiomic, and genetic data performed well. This promising outcome encourages further research using multimodal data models [145]. With the advancement and integration of radiomics and genomics, radiogenomics has increasingly been employed to predict clinical outcomes. Research has demonstrated that radiogenomics can effectively forecast chemotherapy responses and facilitate patient risk stratification. The convergence of multi-omics approaches with radiomics is emerging as a significant trend, warranting application in additional areas such as disease progression prediction to enhance clinical decision-making.

## Conclusions and challenges

Radiomics significantly enhances the accuracy of OC diagnosis and reduces the variability in diagnostic proficiency among physicians. Additionally, it enables precise subtyping of OC without necessitating needle biopsies. Furthermore, radiomics plays a crucial role in predicting survival rates, therapeutic outcomes, tumor metastasis, and recurrence in OC patients, which is of great significance for guiding personalized medicine. In recent years, radiogenomics has emerged as a promising field capable of identifying genetic abnormalities such as *BRCA* mutations. Currently, genetic data are combined with radiomics features to predict clinical outcomes like chemotherapy response and risk stratification that further guide clinical decision-making.

In contrast to other tumor types, the application of image-based AI in OC has been relatively delayed. This discrepancy may be attributed to the fact that radiomics was initially applied and yielded more results in tumors for which CT is the recommended examination technique, such as head and neck tumors and lung cancers [18]. Additionally, we have noted a limited utilization of radiomics in clinical settings for grading OC, despite its more widespread application in head and neck cancers [146, 147]. Currently, numerous researchers are concentrating on predicting OC subtypes to tailor treatment strategies accordingly, as different OC subtypes exhibit significantly varied responses to chemotherapy. While several studies have been conducted to predict the response to targeted therapy and immunotherapy across various tumor types like non-small cell lung cancer, breast cancer, and hepatocellular carcinoma [148–150], similar investigations for OC have not yet been undertaken, as these treatments are not considered standard. However, radiomics has frequently been employed to predict responses to residual disease and chemotherapy in OC, facilitating personalized treatment adjustments that lead to improved patient outcomes. Furthermore, radiomics holds the potential to assist clinicians in making precise diagnoses of both benign and malignant tumors, thereby minimizing delays in disease management and preventing overtreatment of benign tumors before surgery. It can also aid in determining the frequency of imaging follow-ups and postoperative treatment based on predictions regarding patient survival and recurrence rates, ultimately contributing to a significant improvement in survival rates among patients with OC. Moreover, radiogenomic detection of *BRCA* gene mutations provides convenience for the application of targeted therapy by avoiding invasive procedures and reducing associated costs.

Despite these promising outcomes in using radiomics for OC management, several constraints have impeded its widespread adoption in clinical settings. Firstly, the lack of reproducibility in radiomics research presents a significant challenge. The processes used in radiomics vary greatly across different platforms and studies, encompassing aspects from image acquisition to the extraction of multiple features. Factors such as acquisition parameters, reconstruction techniques, scanner manufacturers, and scanning protocols may introduce variability during image acquisition. Secondly, numerous segmentation methods are documented in the literature, including those based on 2D or 3D images, as well as solid, cystic, or whole tumors. The diversity of these methods significantly influences the radiomics features and the adaptability of models constructed using these features. Therefore, standardization of process is crucial for improving reproducibility when conducting radiomics research. Thirdly, despite an increasing number of multi-omics studies focusing on genomics, transcriptomics, and proteomics, there remains a paucity of studies that integrate radiomics with other omics disciplines. The integration of multiple omics has the potential to improve patient survival rates and promote more precise medical approaches in the future. Additionally, the establishment of large and annotated datasets through international collaboration such as the Cancer Imaging Archive will play a supportive role [151]. Moreover, the algorithms utilized in radiomics and radiogenomics are often described as “black boxes”, which may face challenges in the context of AI-enabled imaging biomarkers for optimizing therapy, due to the necessity for biomarker-driven treatment decisions to be grounded in pathophysiological explanations [152]. Therefore, we encourage future studies to prioritize the interpretability of AI models in their modeling efforts to facilitate the translation of benefits into practical applications. Furthermore, it is essential to conduct large sample, multi-center prospective studies to further validate these models and enhance their clinical usability. Researchers should also expand the practical application of radiomics and radiogenomics in clinical practice by synthesizing experiences and addressing shortcomings to improve model performance. Finally, there are several promising fields worth exploring, such as virtual biopsy and delta radiomics. Virtual biopsy enables the acquisition of multi-site tissue information, while delta radiomics introduces a temporal dimension by involving quantitative feature extraction from image sets obtained during treatment [153, 154], thereby providing insights into the evolution of feature values.

In conclusion, through these development directions, radiomics and radiogenomics are expected to become more mature and refined in the future, offering more effective and reliable support for early diagnosis, accurate treatment, and prognostic evaluation of OC.

## Data Availability

The data and materials are all available from this review.

## Abbreviations

2D: Two-dimensional
3D: Three-dimensional
ADC: Apparent diffusion coefficient
AI: Artificial intelligence
AUC: Area under the ROC curve
BOTs: Borderline ovarian tumors
CA125: Cancer antigen 125
CDK2: Cyclin-dependent kinase 2
CE: Contrast-enhanced
CE-T1WI: Contrast-enhanced-T1-weighted imaging
C-index: Concordance indexes
CT: Computed tomography
ctDNA: Circulating tumor DNA
DL: Deep learning
DPS: Delayed primary surgery
DWI: Diffusion weighted imaging
EOC: Epithelial ovarian cancer
ESUR: European society of urogenital radiology
FIGO: Federation of international of gynecologists and obstetricians
FS: Fat-suppressed
HE4: Human epididymis protein 4
HGSOC: High-grade serous ovarian carcinoma
ICA: Independent component analysis
IDS: Interval tumor reduction surgery
ISM: Intersite similarity matrix
LASSO: Least absolute shrinkage and selection operator
LGSOC: Low-grade serous ovarian carcinoma
MAP: Metastases in abdomen and pelvis
MF: Maximal volume of interest and maximal feature value
miRNA: MicroRNA
ML: Machine learning
MRI: Magnetic resonance imaging
mRMR: Minimum redundancy maximum relevance
NAC: Neoadjuvant chemotherapy
NCCN: National comprehensive cancer network
NCI: National cancer institute
NEOC: Non-epithelial ovarian cancer
NGS: Next-generation sequencing
NPV: Negative predictive value
NRI: Networked readiness index
OC: Ovarian cancer
OCCC: Ovarian clear cell carcinoma
O-RADS: Ovarian-adnexal reporting and data system
OS: Overall survival
PARP: Poly ADP-ribose polymerase
PCA: Principal component analysis
PDS: Primary debulking surgery
PET: Positron emission tomography
PFS: Progression-free survival
PM: Peritoneal metastasis
PRISMA: Reporting items for systematic reviews and meta-analyses
RECIST 1.1: Response evaluation criteria in solid tumors
RFE: Recursive feature elimination
RMI: Risk of malignancy index
ROC: Receiver operating characteristic
ROI: Regions of interest
SCST: Sex cord-stromal tumor
SNPs: Single-nucleotide polymorphisms
SULF1: Sulfatase 1
SVM: Support vector machine
T2WI: T2-weighted imaging
TVUS: Transvaginal ultrasound
Vit: Vision transformer
WHO: World Health Organization

## References

[1] Bray F, Laversanne M, Sung H, Ferlay J, Siegel RL, Soerjomataram I, et al. Global cancer statistics 2022: GLOBOCAN estimates of incidence and mortality worldwide for 36 cancers in 185 countries. CA Cancer J Clin. 2024;74(3):229–63. 38572751 10.3322/caac.21834

[2] Cree IA, White VA, Indave BI, Lokuhetty D. Revising the WHO classification: female genital tract tumours. Histopathology. 2020;76(1):151–6. 31846528 10.1111/his.13977

[3] Kossaï M, Leary A, Scoazec JY, Genestie C. Ovarian cancer: a heterogeneous disease. Pathobiology. 2018;85(1–2):41–9. 29020678 10.1159/000479006

[4] Matulonis UA, Sood AK, Fallowfield L, Howitt BE, Sehouli J, Karlan BY. Ovarian cancer. Nat Rev Dis Primers. 2016;2:16061. 27558151 10.1038/nrdp.2016.61 PMC7290868

[5] Panico C, Avesani G, Zormpas-Petridis K, Rundo L, Nero C, Sala E. Radiomics and radiogenomics of ovarian cancer: implications for treatment monitoring and clinical management. Radiol Clin North Am. 2023;61(4):749–60. 37169435 10.1016/j.rcl.2023.02.006

[6] Nougaret S, Tardieu M, Vargas HA, Reinhold C, Vande Perre S, Bonanno N, et al. Ovarian cancer: an update on imaging in the era of radiomics. Diagn Interv Imaging. 2019;100(10):647–55. 30555018 10.1016/j.diii.2018.11.007

[7] Konstantinopoulos PA, Matulonis UA. Clinical and translational advances in ovarian cancer therapy. Nat Cancer. 2023;4(9):1239–57. 37653142 10.1038/s43018-023-00617-9

[8] European Society of Radiology (ESR) (2015) Medical imaging in personalised medicine: a white paper of the research committee of the European Society of Radiology (ESR). Insights Imaging. 2015;6(2):141–55. 10.1007/s13244-015-0394-0 PMC4376812 25763994

[9] Mukama T, Fortner RT, Katzke V, Hynes LC, Petrera A, Hauck SM, et al. Prospective evaluation of 92 serum protein biomarkers for early detection of ovarian cancer. Br J Cancer. 2022;126(9):1301–9. 35031764 10.1038/s41416-021-01697-z PMC9042845

[10] Dochez V, Caillon H, Vaucel E, Dimet J, Winer N, Ducarme G. Biomarkers and algorithms for diagnosis of ovarian cancer: CA125, HE4, RMI and ROMA, a review. J Ovarian Res. 2019;12(1):28. 30917847 10.1186/s13048-019-0503-7 PMC6436208

[11] Zhang YP, Zhang XY, Cheng YT, Li B, Teng XZ, Zhang J, et al. Artificial intelligence-driven radiomics study in cancer: the role of feature engineering and modeling. Mil Med Res. 2023;10(1):22. 37189155 10.1186/s40779-023-00458-8 PMC10186733

[12] Nougaret S, McCague C, Tibermacine H, Vargas HA, Rizzo S, Sala E. Radiomics and radiogenomics in ovarian cancer: a literature review. Abdom Radiol (NY). 2021;46(6):2308–22. 33174120 10.1007/s00261-020-02820-z

[13] Lambin P, Rios-Velazquez E, Leijenaar R, Carvalho S, van Stiphout RG, Granton P, et al. Radiomics: extracting more information from medical images using advanced feature analysis. Eur J Cancer. 2012;48(4):441–6. 22257792 10.1016/j.ejca.2011.11.036 PMC4533986

[14] Kumar V, Gu Y, Basu S, Berglund A, Eschrich SA, Schabath MB, et al. Radiomics: the process and the challenges. Magn Reson Imaging. 2012;30(9):1234–48. 22898692 10.1016/j.mri.2012.06.010 PMC3563280

[15] Aerts HJ, Velazquez ER, Leijenaar RT, Parmar C, Grossmann P, Carvalho S, et al. Decoding tumour phenotype by noninvasive imaging using a quantitative radiomics approach. Nat Commun. 2014;5:4006. 24892406 10.1038/ncomms5006 PMC4059926

[16] Gillies RJ, Kinahan PE, Hricak H. Radiomics: images are more than pictures, they are data. Radiology. 2016;278(2):563–77. 26579733 10.1148/radiol.2015151169 PMC4734157

[17] Huynh BQ, Li H, Giger ML. Digital mammographic tumor classification using transfer learning from deep convolutional neural networks. J Med Imaging (Bellingham). 2016;3(3):034501. 27610399 10.1117/1.JMI.3.3.034501 PMC4992049

[18] Lambin P, Leijenaar RTH, Deist TM, Peerlings J, de Jong EEC, van Timmeren J, et al. Radiomics: the bridge between medical imaging and personalized medicine. Nat Rev Clin Oncol. 2017;14:749–62. 28975929 10.1038/nrclinonc.2017.141

[19] Scapicchio C, Gabelloni M, Barucci A, Cioni D, Saba L, Neri E. A deep look into radiomics. Radiol Med. 2021;126(10):1296–311. 34213702 10.1007/s11547-021-01389-x PMC8520512

[20] Choi RY, Coyner AS, Kalpathy-Cramer J, Chiang MF, Campbell JP. Introduction to machine learning, neural networks, and deep learning. Transl Vis Sci Technol. 2020;9(2):14. 32704420 10.1167/tvst.9.2.14 PMC7347027

[21] Nougaret S, Tibermacine H, Tardieu M, Sala E. Radiomics: an introductory guide to what it may foretell. Curr Oncol Rep. 2019;21(8):70. 31240403 10.1007/s11912-019-0815-1

[22] van Heeswijk MM, Lambregts DMJ, van Griethuysen JJM, Oei S, Rao SX, de Graaff CAM, et al. Automated and semiautomated segmentation of rectal tumor volumes on diffusion-weighted MRI: can it replace manual volumetry?. Int J Radiat Oncol Biol Phys. 2016;94(4):824–31. 26972655 10.1016/j.ijrobp.2015.12.017

[23] Fung YL, Ng KET, Vogrin SJ, Meade C, Ngo M, Collins SJ, et al. Comparative utility of manual versus automated segmentation of hippocampus and entorhinal cortex volumes in a memory clinic sample. J Alzheimers Dis. 2019;68(1):159–71. 30814357 10.3233/JAD-181172

[24] de Sitter A, Verhoeven T, Burggraaff J, Liu Y, Simoes J, Ruggieri S, et al. Reduced accuracy of MRI deep grey matter segmentation in multiple sclerosis: an evaluation of four automated methods against manual reference segmentations in a multi-center cohort. J Neurol. 2020;267(12):3541–54. 32621103 10.1007/s00415-020-10023-1 PMC7674567

[25] Smits LP, van Wijk DF, Duivenvoorden R, Xu D, Yuan C, Stroes ES, et al. Manual versus automated carotid artery plaque component segmentation in high and lower quality 3.0 tesla MRI scans. PLoS One. 2016;11(12):e0164267 10.1371/journal.pone.0164267 PMC5145140 27930665

[26] Mohammad-Rahimi H, Rokhshad R, Bencharit S, Krois J, Schwendicke F. Deep learning: a primer for dentists and dental researchers. J Dent. 2023;130:104430. 36682721 10.1016/j.jdent.2023.104430

[27] Chicklore S, Goh V, Siddique M, Roy A, Marsden PK, Cook GJ. Quantifying tumour heterogeneity in ^18^F-FDG PET/CT imaging by texture analysis. Eur J Nucl Med Mol Imaging. 2013;40(1):133–40. 23064544 10.1007/s00259-012-2247-0

[28] Parekh V, Jacobs MA. Radiomics: a new application from established techniques. Expert Rev Precis Med Drug Dev. 2016;1(2):207–26. 28042608 10.1080/23808993.2016.1164013 PMC5193485

[29] Scalco E, Rizzo G. Texture analysis of medical images for radiotherapy applications. Br J Radiol. 2017;90(1070):20160642. 27885836 10.1259/bjr.20160642 PMC5685100

[30] Andreassen CN, Alsner J, Overgaard J. Does variability in normal tissue reactions after radiotherapy have a genetic basis–where and how to look for it?. Radiother Oncol. 2002;64(2):131–40. 12242122 10.1016/s0167-8140(02)00154-8

[31] Segal E, Sirlin CB, Ooi C, Adler AS, Gollub J, Chen X, et al. Decoding global gene expression programs in liver cancer by noninvasive imaging. Nat Biotechnol. 2007;25(6):675–80. 17515910 10.1038/nbt1306

[32] Bodalal Z, Trebeschi S, Nguyen-Kim TDL, Schats W, Beets-Tan R. Radiogenomics: bridging imaging and genomics. Abdom Radiol (NY). 2019;44(6):1960–84. 31049614 10.1007/s00261-019-02028-w

[33] Cen X, Dong W, Lv W, Zhao Y, Dubee F, Mentis AFA, et al. Towards interpretable imaging genomics analysis: methodological developments and applications. Inf Fusion. 2024;102:102032

[34] Froyman W, Landolfo C, De Cock B, Wynants L, Sladkevicius P, Testa AC, et al. Risk of complications in patients with conservatively managed ovarian tumours (IOTA5): a 2-year interim analysis of a multicentre, prospective, cohort study. Lancet Oncol. 2019;20(3):448–58. 30737137 10.1016/S1470-2045(18)30837-4

[35] Kuroki L, Guntupalli SR. Treatment of epithelial ovarian cancer. BMJ. 2020;371:m3773. 33168565 10.1136/bmj.m3773

[36] Wu M, Wang Y, Su M, Wang R, Sun X, Zhang R, et al. Integrating contrast-enhanced US to O-RADS US for classification of adnexal lesions with solid components: time-intensity curve analysis versus visual assessment. Radiol Imaging Cancer. 2024;6(6):e240024. 39392388 10.1148/rycan.240024 PMC11615631

[37] Stein EB, Wasnik AP, Sciallis AP, Kamaya A, Maturen KE. MR imaging-pathologic correlation in ovarian cancer. Magn Reson Imaging Clin N Am. 2017;25(3):545–62. 28668159 10.1016/j.mric.2017.03.004

[38] Faschingbauer F, Beckmann MW, Weyert Goecke T, Renner S, Häberle L, Benz M, et al. Automatic texture-based analysis in ultrasound imaging of ovarian masses. Ultraschall Med. 2013;34(2):145–50. 22623132 10.1055/s-0031-1299331

[39] Aramendía-Vidaurreta V, Cabeza R, Villanueva A, Navallas J, Alcázar JL. Ultrasound image discrimination between benign and malignant adnexal masses based on a neural network approach. Ultrasound Med Biol. 2016;42(3):742–52. 26715189 10.1016/j.ultrasmedbio.2015.11.014

[40] Zhang H, Mao Y, Chen X, Wu G, Liu X, Zhang P, et al. Magnetic resonance imaging radiomics in categorizing ovarian masses and predicting clinical outcome: a preliminary study. Eur Radiol. 2019;29(7):3358–71. 30963272 10.1007/s00330-019-06124-9

[41] Li J, Zhang T, Ma J, Zhang N, Zhang Z, Ye Z. Machine-learning-based contrast-enhanced computed tomography radiomic analysis for categorization of ovarian tumors. Front Oncol. 2022;12:934735. 36016613 10.3389/fonc.2022.934735 PMC9395674

[42] Hauptmann S, Friedrich K, Redline R, Avril S. Ovarian borderline tumors in the 2014 WHO classification: evolving concepts and diagnostic criteria. Virchows Arch. 2017;470(2):125–42. 28025670 10.1007/s00428-016-2040-8 PMC5298321

[43] Liu X, Wang T, Zhang G, Hua K, Jiang H, Duan S, et al. Two-dimensional and three-dimensional T2 weighted imaging-based radiomic signatures for the preoperative discrimination of ovarian borderline tumors and malignant tumors. J Ovarian Res. 2022;15(1):22. 35115022 10.1186/s13048-022-00943-z PMC8815217

[44] Wei M, Zhang Y, Bai G, Ding C, Xu H, Dai Y, et al. T2-weighted MRI-based radiomics for discriminating between benign and borderline epithelial ovarian tumors: a multicenter study. Insights Imaging. 2022;13(1):130. 35943620 10.1186/s13244-022-01264-x PMC9363551

[45] Qi L, Chen D, Li C, Li J, Wang J, Zhang C, et al. Diagnosis of ovarian neoplasms using nomogram in combination with ultrasound image-based radiomics signature and clinical factors. Front Genet. 2021;12:753948. 34650603 10.3389/fgene.2021.753948 PMC8505695

[46] Ratnavelu ND, Brown AP, Mallett S, Scholten RJ, Patel A, Founta C, et al. Intraoperative frozen section analysis for the diagnosis of early stage ovarian cancer in suspicious pelvic masses. Cochrane Database Syst Rev. 2016;3(3):CD010360. 10.1002/14651858.CD010360.pub2 PMC6457848 26930463

[47] van Nagell JR Jr, Burgess BT, Miller RW, Baldwin L, DeSimone CP, Ueland FR, et al. Survival of women with type I and II epithelial ovarian cancer detected by ultrasound screening. Obstet Gynecol. 2018;132(5):1091–100 10.1097/AOG.0000000000002921 PMC9373050 30303916

[48] Yu H, Wang J, Wu B, Li J, Chen R. Prognostic significance and risk factors for pelvic and para-aortic lymph node metastasis in type I and type II ovarian cancer: a large population-based database analysis. J Ovarian Res. 2023;16(1):28. 36717897 10.1186/s13048-023-01102-8 PMC9885671

[49] Meinhold-Heerlein I, Fotopoulou C, Harter P, Kurzeder C, Mustea A, Wimberger P, et al. The new WHO classification of ovarian, fallopian tube, and primary peritoneal cancer and its clinical implications. Arch Gynecol Obstet. 2016;293(4):695–700. 26894303 10.1007/s00404-016-4035-8

[50] Kurman RJ, Shih IM. The dualistic model of ovarian carcinogenesis: revisited, revised, and expanded. Am J Pathol. 2016;186(4):733–47. 27012190 10.1016/j.ajpath.2015.11.011 PMC5808151

[51] Qian L, Ren J, Liu A, Gao Y, Hao F, Zhao L, et al. MR imaging of epithelial ovarian cancer: a combined model to predict histologic subtypes. Eur Radiol. 2020;30(11):5815–25. 32535738 10.1007/s00330-020-06993-5

[52] Jian J, Li Y, Pickhardt PJ, Xia W, He Z, Zhang R, et al. MR image-based radiomics to differentiate type I and type II epithelial ovarian cancers. Eur Radiol. 2021;31(1):403–10. 32743768 10.1007/s00330-020-07091-2

[53] Xu Y, Luo HJ, Ren J, Guo LM, Niu J, Song X. Diffusion-weighted imaging-based radiomics in epithelial ovarian tumors: assessment of histologic subtype. Front Oncol. 2022;12:978123. 36544703 10.3389/fonc.2022.978123 PMC9762272

[54] Yao F, Ding J, Lin F, Xu X, Jiang Q, Zhang L, et al. Nomogram based on ultrasound radiomics score and clinical variables for predicting histologic subtypes of epithelial ovarian cancer. Br J Radiol. 2022;95(1136):20211332. 35612547 10.1259/bjr.20211332 PMC10162053

[55] Tang ZP, Ma Z, He Y, Liu RC, Jin BB, Wen DY, et al. Ultrasound-based radiomics for predicting different pathological subtypes of epithelial ovarian cancer before surgery. BMC Med Imaging. 2022;22(1):147. 35996097 10.1186/s12880-022-00879-2 PMC9396799

[56] Li J, Li X, Ma J, Wang F, Cui S, Ye Z. Computed tomography-based radiomics machine learning classifiers to differentiate type I and type II epithelial ovarian cancers. Eur Radiol. 2023;33(7):5193–204. 36515713 10.1007/s00330-022-09318-w

[57] Kim J, Park EY, Kim O, Schilder JM, Coffey DM, Cho CH, et al. Cell origins of high-grade serous ovarian cancer. Cancers (Basel). 2018;10(11):433. 30424539 10.3390/cancers10110433 PMC6267333

[58] Chung YS, Lee JY, Kim HS, Nam EJ, Kim SW, Kim YT. Outcomes of non-high grade serous carcinoma after neoadjuvant chemotherapy for advanced-stage ovarian cancer: single-institution experience. Yonsei Med J. 2018;59(8):930–6. 30187699 10.3349/ymj.2018.59.8.930 PMC6127428

[59] An H, Wang Y, Wong EMF, Lyu S, Han L, Perucho JAU, et al. CT texture analysis in histological classification of epithelial ovarian carcinoma. Eur Radiol. 2021;31(7):5050–8. 33409777 10.1007/s00330-020-07565-3

[60] Wang M, Perucho JAU, Hu Y, Choi MH, Han L, Wong EMF, et al. Computed tomographic radiomics in differentiating histologic subtypes of epithelial ovarian carcinoma. JAMA Netw Open. 2022;5(12):e2245141. 36469315 10.1001/jamanetworkopen.2022.45141 PMC9855300

[61] Li C, Wang H, Chen Y, Zhu C, Gao Y, Wang X, et al. Nomograms of combining MRI multisequences radiomics and clinical factors for differentiating high-grade from low-grade serous ovarian carcinoma. Front Oncol. 2022;12:816982. 35747838 10.3389/fonc.2022.816982 PMC9211758

[62] Zhu H, Ai Y, Zhang J, Zhang J, Jin J, Xie C, et al. Preoperative nomogram for differentiation of histological subtypes in ovarian cancer based on computer tomography radiomics. Front Oncol. 2021;11:642892. 33842352 10.3389/fonc.2021.642892 PMC8027335

[63] Cheng M, Tan S, Ren T, Zhu Z, Wang K, Zhang L, et al. Magnetic resonance imaging radiomics to differentiate ovarian sex cord-stromal tumors and primary epithelial ovarian cancers. Front Oncol. 2022;12:1073983. 36713500 10.3389/fonc.2022.1073983 PMC9880468

[64] Ren J, Mao L, Zhao J, Li XL, Wang C, Liu XY, et al. Seeing beyond the tumor: computed tomography image-based radiomic analysis helps identify ovarian clear cell carcinoma subtype in epithelial ovarian cancer. Radiol Med. 2023;128(8):900–11. 37368228 10.1007/s11547-023-01666-x

[65] Gonzalez Bosquet J, Devor EJ, Newtson AM, Smith BJ, Bender DP, Goodheart MJ, et al. Creation and validation of models to predict response to primary treatment in serous ovarian cancer. Sci Rep. 2021;11(1):5957. 33727600 10.1038/s41598-021-85256-9 PMC7971042

[66] Hong Y, Liu Z, Lin D, Peng J, Yuan Q, Zeng Y, et al. Development of a radiomic-clinical nomogram for prediction of survival in patients with serous ovarian cancer. Clin Radiol. 2022;77(5):352–9. 35264303 10.1016/j.crad.2022.01.038

[67] Zheng Y, Wang F, Zhang W, Li Y, Yang B, Yang X, et al. Preoperative CT-based deep learning model for predicting overall survival in patients with high-grade serous ovarian cancer. Front Oncol. 2022;12:986089. 36158664 10.3389/fonc.2022.986089 PMC9504666

[68] Lu H, Arshad M, Thornton A, Avesani G, Cunnea P, Curry E, et al. A mathematical-descriptor of tumor-mesoscopic-structure from computed-tomography images annotates prognostic- and molecular-phenotypes of epithelial ovarian cancer. Nat Commun. 2019;10(1):764. 30770825 10.1038/s41467-019-08718-9 PMC6377605

[69] Fotopoulou C, Rockall A, Lu H, Lee P, Avesani G, Russo L, et al. Validation analysis of the novel imaging-based prognostic radiomic signature in patients undergoing primary surgery for advanced high-grade serous ovarian cancer (HGSOC). Br J Cancer. 2022;126(7):1047–54. 34923575 10.1038/s41416-021-01662-w PMC8979975

[70] Bagnoli M, Canevari S, Califano D, Losito S, Maio MD, Raspagliesi F, et al. Development and validation of a microRNA-based signature (MiROvaR) to predict early relapse or progression of epithelial ovarian cancer: a cohort study. Lancet Oncol. 2016;17(8):1137–46. 27402147 10.1016/S1470-2045(16)30108-5

[71] Ladanyi A, Mukherjee A, Kenny HA, Johnson A, Mitra AK, Sundaresan S, et al. Adipocyte-induced CD36 expression drives ovarian cancer progression and metastasis. Oncogene. 2018;37(17):2285–301. 29398710 10.1038/s41388-017-0093-z PMC5920730

[72] Bryant A, Hiu S, Kunonga PT, Gajjar K, Craig D, Vale L, et al. Impact of residual disease as a prognostic factor for survival in women with advanced epithelial ovarian cancer after primary surgery. Cochrane Database Syst Rev. 2022;9(9):CD015048. 10.1002/14651858.CD015048.pub2 PMC9512080 36161421

[73] Chase DM, Mahajan A, Scott DA, Hawkins N, Kalilani L. The impact of varying levels of residual disease following cytoreductive surgery on survival outcomes in patients with ovarian cancer: a meta-analysis. BMC Womens Health. 2024;24(1):179. 38491366 10.1186/s12905-024-02977-5 PMC10941390

[74] Coleridge SL, Bryant A, Kehoe S, Morrison J. Neoadjuvant chemotherapy before surgery versus surgery followed by chemotherapy for initial treatment in advanced ovarian epithelial cancer. Cochrane Database Syst Rev. 2021;7(7):CD005343. 10.1002/14651858.CD005343.pub6 PMC8406953 34328210

[75] Chi DS, Eisenhauer EL, Lang J, Huh J, Haddad L, Abu-Rustum NR, et al. What is the optimal goal of primary cytoreductive surgery for bulky stage IIIC epithelial ovarian carcinoma (EOC)?. Gynecol Oncol. 2006;103(2):559–64. 16714056 10.1016/j.ygyno.2006.03.051

[76] Cho JH, Kim S, Song YS. Neoadjuvant chemotherapy in advanced ovarian cancer: optimal patient selection and response evaluation. Chin Clin Oncol. 2018;7(6):58. 30509079 10.21037/cco.2018.10.11

[77] Kobal B, Noventa M, Cvjeticanin B, Barbic M, Meglic L, Herzog M, et al. Primary debulking surgery versus primary neoadjuvant chemotherapy for high grade advanced stage ovarian cancer: comparison of survivals. Radiol Oncol. 2018;52(3):307–19. 30210049 10.2478/raon-2018-0030 PMC6137361

[78] Ghirardi V, Moruzzi MC, Bizzarri N, Vargiu V, D’Indinosante M, Garganese G, et al. Minimal residual disease at primary debulking surgery versus complete tumor resection at interval debulking surgery in advanced epithelial ovarian cancer: a survival analysis. Gynecol Oncol. 2020;157(1):209–13. 31952843 10.1016/j.ygyno.2020.01.010

[79] Miceli V, Gennarini M, Tomao F, Cupertino A, Lombardo D, Palaia I, et al. Imaging of peritoneal carcinomatosis in advanced ovarian cancer: CT, MRI, radiomic features and resectability criteria. Cancers (Basel). 2023;15(24):5827. 38136373 10.3390/cancers15245827 PMC10741537

[80] van de Vrie R, Rutten MJ, Asseler JD, Leeflang MM, Kenter GG, Mol BWJ, et al. Laparoscopy for diagnosing resectability of disease in women with advanced ovarian cancer. Cochrane Database Syst Rev. 2019;3(3):CD009786. 10.1002/14651858.CD009786.pub3 PMC6432174 30907434

[81] Li H, Zhang R, Li R, Xia W, Chen X, Zhang J, et al. Noninvasive prediction of residual disease for advanced high-grade serous ovarian carcinoma by MRI-based radiomic-clinical nomogram. Eur Radiol. 2021;31(10):7855–64. 33864139 10.1007/s00330-021-07902-0

[82] Lu J, Cai S, Wang F, Wu PY, Pan X, Qiang J, et al. Development of a prediction model for gross residual in high-grade serous ovarian cancer by combining preoperative assessments of abdominal and pelvic metastases and multiparametric MRI. Acad Radiol. 2023;30(9):1823–31. 36587996 10.1016/j.acra.2022.12.019

[83] Rizzo S, Botta F, Raimondi S, Origgi D, Buscarino V, Colarieti A, et al. Radiomics of high-grade serous ovarian cancer: association between quantitative CT features, residual tumour and disease progression within 12 months. Eur Radiol. 2018;28(11):4849–59. 29737390 10.1007/s00330-018-5389-z

[84] Cohen PA, Powell A, Böhm S, Gilks CB, Stewart CJR, Meniawy TM, et al. Pathological chemotherapy response score is prognostic in tubo-ovarian high-grade serous carcinoma: a systematic review and meta-analysis of individual patient data. Gynecol Oncol. 2019;154(2):441–8. 31118141 10.1016/j.ygyno.2019.04.679

[85] Rundo L, Beer L, Escudero Sanchez L, Crispin-Ortuzar M, Reinius M, McCague C, et al. Clinically interpretable radiomics-based prediction of histopathologic response to neoadjuvant chemotherapy in high-grade serous ovarian carcinoma. Front Oncol. 2022;12:868265. 35785153 10.3389/fonc.2022.868265 PMC9243357

[86] Li H, Cai S, Deng L, Xiao Z, Guo Q, Qiang J, et al. Prediction of platinum resistance for advanced high-grade serous ovarian carcinoma using MRI-based radiomics nomogram. Eur Radiol. 2023;33(8):5298–308. 36995415 10.1007/s00330-023-09552-w

[87] Konstantinopoulos PA, Waggoner S, Vidal GA, Mita M, Moroney JW, Holloway R, et al. Single-arm phases 1 and 2 trial of niraparib in combination with pembrolizumab in patients with recurrent platinum-resistant ovarian carcinoma. JAMA Oncol. 2019;5(8):1141–9. 31194228 10.1001/jamaoncol.2019.1048 PMC6567832

[88] Konstantinopoulos PA, Cheng SC, Wahner Hendrickson AE, Penson RT, Schumer ST, Doyle LA, et al. Berzosertib plus gemcitabine versus gemcitabine alone in platinum-resistant high-grade serous ovarian cancer: a multicentre, open-label, randomised, phase 2 trial. Lancet Oncol. 2020;21(7):957–68. 32553118 10.1016/S1470-2045(20)30180-7 PMC8023719

[89] Lheureux S, Gourley C, Vergote I, Oza AM. Epithelial ovarian cancer. Lancet. 2019;393(10177):1240–53. 30910306 10.1016/S0140-6736(18)32552-2

[90] Kurnit KC, Fleming GF, Lengyel E. Updates and new options in advanced epithelial ovarian cancer treatment. Obstet Gynecol. 2021;137(1):108–21. 33278287 10.1097/AOG.0000000000004173 PMC7737875

[91] Forstner R. Early detection of ovarian cancer. Eur Radiol. 2020;30(10):5370–3. 32468105 10.1007/s00330-020-06937-z PMC7476911

[92] Ai Y, Zhang J, Jin J, Zhang J, Zhu H, Jin X. Preoperative prediction of metastasis for ovarian cancer based on computed tomography radiomics features and clinical factors. Front Oncol. 2021;11:610742. 34178617 10.3389/fonc.2021.610742 PMC8222738

[93] Matsuo K, Klar M, Barakzai SK, Jooya ND, Nusbaum DJ, Shimada M, et al. Utilization of sentinel lymph node biopsy in the early ovarian cancer surgery. Arch Gynecol Obstet. 2023;307(2):525–32. 35595998 10.1007/s00404-022-06595-0

[94] Chalif J, Yao M, Gruner M, Kuznicki M, Vargas R, Rose PG, et al. Incidence and prognostic significance of inguinal lymph node metastasis in women with newly diagnosed epithelial ovarian cancer. Gynecol Oncol. 2022;165(1):90–6. 35272875 10.1016/j.ygyno.2022.01.026

[95] Wang F, Wang Y, Zhou Y, Liu C, Liang D, Xie L, et al. Apparent diffusion coefficient histogram analysis for assessing tumor staging and detection of lymph node metastasis in epithelial ovarian cancer: correlation with p53 and Ki-67 expression. Mol Imaging Biol. 2019;21(4):731–9. 30456593 10.1007/s11307-018-1295-7

[96] Zhou J, Sun JY, Wu SG, Wang X, He ZY, Chen QH, et al. Risk factors for lymph node metastasis in ovarian cancer: implications for systematic lymphadenectomy. Int J Surg. 2016;29:123–7. 27000718 10.1016/j.ijsu.2016.03.039

[97] Yuan Y, Gu ZX, Tao XF, Liu SY. Computer tomography, magnetic resonance imaging, and positron emission tomography or positron emission tomography/computer tomography for detection of metastatic lymph nodes in patients with ovarian cancer: a meta-analysis. Eur J Radiol. 2012;81(5):1002–6. 21349672 10.1016/j.ejrad.2011.01.112

[98] Erdem B, Yüksel IT, Peker N, Ulukent SC, Aşıcıoğlu O, Özaydin IY, et al. Evaluation of factors affecting lymph node metastasis in clinical stage I-II epithelial ovarian cancer. Oncol Res Treat. 2018;41(7–8):444–8. 29975960 10.1159/000488082

[99] Benson AB, Venook AP, Al-Hawary MM, Arain MA, Chen YJ, Ciombor KK, et al. Colon cancer, version 2. 2021, NCCN clinical practice guidelines in oncology. J Natl Compr Canc Netw. 2021;19(3):329–59. 10.6004/jnccn.2021.0012 33724754

[100] Fischerova D, Burgetova A. Imaging techniques for the evaluation of ovarian cancer. Best Pract Res Clin Obstet Gynaecol. 2014;28(5):697–720. 24846098 10.1016/j.bpobgyn.2014.04.006

[101] Harter P, Gnauert K, Hils R, Lehmann TG, Fisseler-Eckhoff A, Traut A, et al. Pattern and clinical predictors of lymph node metastases in epithelial ovarian cancer. Int J Gynecol Cancer. 2007;17(6):1238–44. 17433064 10.1111/j.1525-1438.2007.00931.x

[102] Chen HZ, Wang XR, Zhao FM, Chen XJ, Li XS, Ning G, et al. The development and validation of a CT-based radiomics nomogram to preoperatively predict lymph node metastasis in high-grade serous ovarian cancer. Front Oncol. 2021;11:711648. 34532289 10.3389/fonc.2021.711648 PMC8438232

[103] Hengeveld EM, Zusterzeel PLM, Lajer H, Høgdall CK, Rosendahl M. The value of surgical staging in patients with apparent early stage epithelial ovarian carcinoma. Gynecol Oncol. 2019;154(2):308–13. 31230820 10.1016/j.ygyno.2019.06.006

[104] Power JW, Dempsey PJ, Yates A, Fenlon H, Mulsow J, Shields C, et al. Peritoneal malignancy: anatomy, pathophysiology and an update on modern day imaging. Br J Radiol. 2022;95(1132):20210217. 34826229 10.1259/bjr.20210217 PMC9153709

[105] Tsili AC, Alexiou G, Tzoumpa M, Siempis T, Argyropoulou MI. Imaging of peritoneal metastases in ovarian cancer using MDCT, MRI, and FDG PET/CT: a systematic review and meta-analysis. Cancers (Basel). 2024;16(8):1467. 38672549 10.3390/cancers16081467 PMC11048266

[106] Song XL, Ren JL, Yao TY, Zhao D, Niu J. Radiomics based on multisequence magnetic resonance imaging for the preoperative prediction of peritoneal metastasis in ovarian cancer. Eur Radiol. 2021;31(11):8438–46. 33948702 10.1007/s00330-021-08004-7

[107] Yu XY, Ren J, Jia Y, Wu H, Niu G, Liu A, et al. Multiparameter MRI radiomics model predicts preoperative peritoneal carcinomatosis in ovarian cancer. Front Oncol. 2021;11:765652. 34790579 10.3389/fonc.2021.765652 PMC8591658

[108] Wei M, Zhang Y, Ding C, Jia J, Xu H, Dai Y, et al. Associating peritoneal metastasis with T2-weighted MRI images in epithelial ovarian cancer using deep learning and radiomics: a multicenter study. J Magn Reson Imaging. 2024;59(1):122–31. 37134000 10.1002/jmri.28761

[109] Armstrong DK, Alvarez RD, Backes FJ, Bakkum-Gamez JN, Barroilhet L, Behbakht K, et al. NCCN guidelines^®^ insights: ovarian cancer, version 3.2022. J Natl Compr Canc Netw. 2022;20(9):972–80. 10.6004/jnccn.2022.0047 36075393

[110] Richardson DL, Sill MW, Coleman RL, Sood AK, Pearl ML, Kehoe SM, et al. Paclitaxel with and without pazopanib for persistent or recurrent ovarian cancer: a randomized clinical trial. JAMA Oncol. 2018;4(2):196–202. 29242937 10.1001/jamaoncol.2017.4218 PMC5838582

[111] Ovarian Tumor Tissue Analysis (OTTA) Consortium, Goode EL, Block MS, Kalli KR, Vierkant RA, Chen W, et al. Dose-response association of CD8^+^ tumor-infiltrating lymphocytes and survival time in high-grade serous ovarian cancer. JAMA Oncol. 2017;3(12):e173290. 10.1001/jamaoncol.2017.3290 PMC5744673 29049607

[112] Tewari KS, Burger RA, Enserro D, Norquist BM, Swisher EM, Brady MF, et al. Final overall survival of a randomized trial of bevacizumab for primary treatment of ovarian cancer. J Clin Oncol. 2019;37(26):2317–28. 31216226 10.1200/JCO.19.01009 PMC6879307

[113] Wang S, Liu Z, Rong Y, Zhou B, Bai Y, Wei W, et al. Deep learning provides a new computed tomography-based prognostic biomarker for recurrence prediction in high-grade serous ovarian cancer. Radiother Oncol. 2019;132:171–7. 30392780 10.1016/j.radonc.2018.10.019

[114] Chen HZ, Wang XR, Zhao FM, Chen XJ, Li XS, Ning G, et al. A CT-based radiomics nomogram for predicting early recurrence in patients with high-grade serous ovarian cancer. Eur J Radiol. 2021;145:110018. 34773830 10.1016/j.ejrad.2021.110018

[115] Wang X, Lu Z. Radiomics analysis of PET and CT components of (18)F-FDG PET/CT imaging for prediction of progression-free survival in advanced high-grade serous ovarian cancer. Front Oncol. 2021;11:638124. 33928029 10.3389/fonc.2021.638124 PMC8078590

[116] Liu L, Wan H, Liu L, Wang J, Tang Y, Cui S, et al. Deep learning provides a new magnetic resonance imaging-based prognostic biomarker for recurrence prediction in high-grade serous ovarian cancer. Diagnostics (Basel). 2023;13(4):748. 36832236 10.3390/diagnostics13040748 PMC9954966

[117] Wang T, Wang H, Wang Y, Liu X, Ling L, Zhang G, et al. MR-based radiomics-clinical nomogram in epithelial ovarian tumor prognosis prediction: tumor body texture analysis across various acquisition protocols. J Ovarian Res. 2022;15(1):6. 35022079 10.1186/s13048-021-00941-7 PMC8753904

[118] Li HM, Gong J, Li RM, Xiao ZB, Qiang JW, Peng WJ, et al. Development of MRI-based radiomics model to predict the risk of recurrence in patients with advanced high-grade serous ovarian carcinoma. AJR Am J Roentgenol. 2021;217(3):664–75. 34259544 10.2214/AJR.20.23195

[119] Yao F, Ding J, Hu Z, Cai M, Liu J, Huang X, et al. Ultrasound-based radiomics score: a potential biomarker for the prediction of progression-free survival in ovarian epithelial cancer. Abdom Radiol (NY). 2021;46(10):4936–45. 34120235 10.1007/s00261-021-03163-z

[120] Tjokrowidjaja A, Friedlander M, Lord SJ, Asher R, Rodrigues M, Ledermann JA, et al. Prognostic nomogram for progression-free survival in patients with BRCA mutations and platinum-sensitive recurrent ovarian cancer on maintenance olaparib therapy following response to chemotherapy. Eur J Cancer. 2021;154:190–200. 34293664 10.1016/j.ejca.2021.06.024

[121] Konstantinopoulos PA, Ceccaldi R, Shapiro GI, D’Andrea AD. Homologous recombination deficiency: exploiting the fundamental vulnerability of ovarian cancer. Cancer Discov. 2015;5(11):1137–54. 26463832 10.1158/2159-8290.CD-15-0714 PMC4631624

[122] Marchetti C, Ataseven B, Perrone AM, Cassani C, Fruscio R, Sassu CM, et al. Clinical characteristics and survival outcome of early-stage, high-grade, serous tubo-ovarian carcinoma according to BRCA mutational status. Gynecol Oncol. 2024;187:170–7. 38788514 10.1016/j.ygyno.2024.05.008

[123] Kim SI, Lee M, Kim HS, Chung HH, Kim JW, Park NH, et al. Effect of BRCA mutational status on survival outcome in advanced-stage high-grade serous ovarian cancer. J Ovarian Res. 2019;12(1):40. 31064392 10.1186/s13048-019-0511-7 PMC6505247

[124] Petrillo M, Marchetti C, De Leo R, Musella A, Capoluongo E, Paris I, et al. BRCA mutational status, initial disease presentation, and clinical outcome in high-grade serous advanced ovarian cancer: a multicenter study. Am J Obstet Gynecol. 2017;217(3):334.e1-e9. 28549976 10.1016/j.ajog.2017.05.036

[125] Nougaret S, Lakhman Y, Gönen M, Goldman DA, Miccò M, D’Anastasi M, et al. High-grade serous ovarian cancer: associations between BRCA mutation status, CT imaging phenotypes, and clinical outcomes. Radiology. 2017;285(2):472–81. 28628421 10.1148/radiol.2017161697 PMC5673044

[126] Tattersall A, Ryan N, Wiggans AJ, Rogozińska E, Morrison J. Poly(ADP-ribose) polymerase (PARP) inhibitors for the treatment of ovarian cancer. Cochrane Database Syst Rev. 2022;2(2):CD007929. 10.1002/14651858.CD007929.pub4 PMC8848772 35170751

[127] Vargas HA, Huang EP, Lakhman Y, Ippolito JE, Bhosale P, Mellnick V, et al. Radiogenomics of high-grade serous ovarian cancer: multireader multi-institutional study from the cancer genome atlas ovarian cancer imaging research group. Radiology. 2017;285(2):482–92. 28641043 10.1148/radiol.2017161870 PMC5673051

[128] Meier A, Veeraraghavan H, Nougaret S, Lakhman Y, Sosa R, Soslow RA, et al. Association between CT-texture-derived tumor heterogeneity, outcomes, and BRCA mutation status in patients with high-grade serous ovarian cancer. Abdom Radiol (NY). 2019;44(6):2040–7. 30474722 10.1007/s00261-018-1840-5 PMC8009104

[129] Mingzhu L, Yaqiong G, Mengru L, Wei W. Prediction of BRCA gene mutation status in epithelial ovarian cancer by radiomics models based on 2D and 3D CT images. BMC Med Imaging. 2021;21(1):180. 34836507 10.1186/s12880-021-00711-3 PMC8626978

[130] Avesani G, Tran HE, Cammarata G, Botta F, Raimondi S, Russo L, et al. CT-based radiomics and deep learning for BRCA mutation and progression-free survival prediction in ovarian cancer using a multicentric dataset. Cancers (Basel). 2022;14(11):2739. 35681720 10.3390/cancers14112739 PMC9179845

[131] Aziz D, Etemadmoghadam D, Caldon CE, Au-Yeung G, Deng N, Hutchinson R, et al. 19q12 amplified and non-amplified subsets of high grade serous ovarian cancer with overexpression of cyclin E1 differ in their molecular drivers and clinical outcomes. Gynecol Oncol. 2018;151(2):327–36. 30209015 10.1016/j.ygyno.2018.08.039

[132] Stronach EA, Paul J, Timms KM, Hughes E, Brown K, Neff C, et al. Biomarker assessment of HR deficiency, tumor BRCA1/2 mutations, and CCNE1 copy number in ovarian cancer: associations with clinical outcome following platinum monotherapy. Mol Cancer Res. 2018;16(7):1103–11. 29724815 10.1158/1541-7786.MCR-18-0034

[133] Lashen A, Algethami M, Alqahtani S, Shoqafi A, Sheha A, Jeyapalan JN, et al. The clinicopathological significance of the cyclin D1/E1-cyclin-dependent kinase (CDK2/4/6)-retinoblastoma (RB1/pRB1) pathway in epithelial ovarian cancers. Int J Mol Sci. 2024;25(7):4060. 38612869 10.3390/ijms25074060 PMC11012085

[134] Fang D, Huang S, Su SB. Cyclin E1-CDK 2, a potential anticancer target. Aging (Albany NY). 2016;8(4):571–2. 27085092 10.18632/aging.100946 PMC4925813

[135] Kanska J, Zakhour M, Taylor-Harding B, Karlan BY, Wiedemeyer WR. Cyclin E as a potential therapeutic target in high grade serous ovarian cancer. Gynecol Oncol. 2016;143(1):152–8. 27461360 10.1016/j.ygyno.2016.07.111

[136] Vargas HA, Veeraraghavan H, Micco M, Nougaret S, Lakhman Y, Meier AA, et al. A novel representation of inter-site tumour heterogeneity from pre-treatment computed tomography textures classifies ovarian cancers by clinical outcome. Eur Radiol. 2017;27(9):3991–4001. 28289945 10.1007/s00330-017-4779-y PMC5545058

[137] Clamp AR, James EC, McNeish IA, Dean A, Kim JW, O’Donnell DM, et al. Weekly dose-dense chemotherapy in first-line epithelial ovarian, fallopian tube, or primary peritoneal carcinoma treatment (ICON8): primary progression free survival analysis results from a GCIG phase 3 randomised controlled trial. Lancet. 2019;394(10214):2084–95. 31791688 10.1016/S0140-6736(19)32259-7 PMC6902268

[138] Tajik P, van de Vrie R, Zafarmand MH, Coens C, Buist MR, Vergote I, et al. The FIGO stage IVA versus IVB of ovarian cancer: prognostic value and predictive value for neoadjuvant chemotherapy. Int J Gynecol Cancer. 2018;28(3):453–8. 29324537 10.1097/IGC.0000000000001186

[139] Zhang D, Jiang YX, Luo SJ, Zhou R, Jiang QX, Linghu H. Serum CA125 levels predict outcome of interval debulking surgery after neoadjuvant chemotherapy in patients with advanced ovarian cancer. Clin Chim Acta. 2018;484:32–5. 29702068 10.1016/j.cca.2018.04.030

[140] Sharbatoghli M, Vafaei S, Aboulkheyr Es H, Asadi-Lari M, Totonchi M, Madjd Z. Prediction of the treatment response in ovarian cancer: a ctDNA approach. J Ovarian Res. 2020;13(1):124. 33076944 10.1186/s13048-020-00729-1 PMC7574472

[141] Nicolasjilwan M, Hu Y, Yan C, Meerzaman D, Holder CA, Gutman D, et al. Addition of MR imaging features and genetic biomarkers strengthens glioblastoma survival prediction in TCGA patients. J Neuroradiol. 2015;42(4):212–21. 24997477 10.1016/j.neurad.2014.02.006 PMC5511631

[142] Grossmann P, Stringfield O, El-Hachem N, Bui MM, Rios Velazquez E, Parmar C, et al. Defining the biological basis of radiomic phenotypes in lung cancer. Elife. 2017;6:e23421. 28731408 10.7554/eLife.23421 PMC5590809

[143] Crispin-Ortuzar M, Woitek R, Reinius MAV, Moore E, Beer L, Bura V, et al. Integrated radiogenomics models predict response to neoadjuvant chemotherapy in high grade serous ovarian cancer. Nat Commun. 2023;14(1):6756. 37875466 10.1038/s41467-023-41820-7 PMC10598212

[144] Yi X, Liu Y, Zhou B, Xiang W, Deng A, Fu Y, et al. Incorporating SULF1 polymorphisms in a pretreatment CT-based radiomic model for predicting platinum resistance in ovarian cancer treatment. Biomed Pharmacother. 2021;133:111013. 33227705 10.1016/j.biopha.2020.111013

[145] Boehm KM, Aherne EA, Ellenson L, Nikolovski I, Alghamdi M, Vázquez-García I, et al. Multimodal data integration using machine learning improves risk stratification of high-grade serous ovarian cancer. Nat Cancer. 2022;3(6):723–33. 35764743 10.1038/s43018-022-00388-9 PMC9239907

[146] Wu W, Ye J, Wang Q, Luo J, Xu S. CT-based radiomics signature for the preoperative discrimination between head and neck squamous cell carcinoma grades. Front Oncol. 2019;9:821. 31544063 10.3389/fonc.2019.00821 PMC6729100

[147] Mukherjee P, Cintra M, Huang C, Zhou M, Zhu S, Colevas AD, et al. CT-based radiomic signatures for predicting histopathologic features in head and neck squamous cell carcinoma. Radiol Imaging Cancer. 2020;2(3):e190039. 32550599 10.1148/rycan.2020190039 PMC7263288

[148] Mehta S, Hughes NP, Li S, Jubb A, Adams R, Lord S, et al. Radiogenomics monitoring in breast cancer identifies metabolism and immune checkpoints as early actionable mechanisms of resistance to anti-angiogenic treatment. EBioMedicine. 2016;10:109–16. 27474395 10.1016/j.ebiom.2016.07.017 PMC5006694

[149] Aerts HJ, Grossmann P, Tan Y, Oxnard GR, Rizvi N, Schwartz LH, et al. Defining a radiomic response phenotype: a pilot study using targeted therapy in NSCLC. Sci Rep. 2016;6:33860. 27645803 10.1038/srep33860 PMC5028716

[150] Sun R, Limkin EJ, Vakalopoulou M, Dercle L, Champiat S, Han SR, et al. A radiomics approach to assess tumour-infiltrating CD8 cells and response to anti-PD-1 or anti-PD-L1 immunotherapy: an imaging biomarker, retrospective multicohort study. Lancet Oncol. 2018;19(9):1180–91. 30120041 10.1016/S1470-2045(18)30413-3

[151] Liu Z, Wang S, Dong D, Wei J, Fang C, Zhou X, et al. The applications of radiomics in precision diagnosis and treatment of oncology: opportunities and challenges. Theranostics. 2019;9(5):1303–22. 30867832 10.7150/thno.30309 PMC6401507

[152] Yang G, Ye Q, Xia J. Unbox the black-box for the medical explainable AI via multi-modal and multi-centre data fusion: a mini-review, two showcases and beyond. Inf Fusion. 2022;77:29–52. 34980946 10.1016/j.inffus.2021.07.016 PMC8459787

[153] Marcu DC, Grava C, Marcu LG. Current role of delta radiomics in head and neck oncology. Int J Mol Sci. 2023;24(3):2214. 36768535 10.3390/ijms24032214 PMC9916410

[154] Gill RR. Virtual image-based biopsy of lung metastases: the promise of radiomics. Acad Radiol. 2023;30(1):47–8. 36371374 10.1016/j.acra.2022.10.030

